# Unveiling Synergistic Effectiveness of Strategically Designed Cobalt Clusters for Efficient Water Electrolysis

**DOI:** 10.1021/acscatal.4c06466

**Published:** 2025-01-27

**Authors:** Abhishikta Chatterjee, Papri Mondal, Priyanka Chakraborty, Sourav Mandal, Corrado Rizzoli, Carlos J. Gómez-García, Bibhutosh Adhikary, Dulal Senapati, Subrata K. Dey

**Affiliations:** † Department of Chemistry, 362966 Sidho-Kanho-Birsha University, Purulia 723104, WB, India; ‡ Department of Chemistry, 30130 Indian Institution of Engineering Science and Technology, Shibpur, Howrah 711103, India; § Dipartimento S.C.V.S.A., 9370 Università di Parma, Parco Area delle Scienze 17/A, Parma I-43124, Italy; ∥ Departamento de Química Inorgánica. Universidad de Valencia, C/Dr. Moliner 50, 46100 Burjasot, Valencia, Spain; ⊥ Chemical Sciences Division, 30176 Saha Institute of Nuclear Physics, 1/AF Bidhannagar, Kolkata 700064, India

**Keywords:** electrocatalysts, water splitting, [2 × 2] Co^II^ grid, heterovalent Co-complex, oxygen evolution reaction (OER), hydrogen evolution reaction (HER)

## Abstract

Electrocatalytic water splitting is a challenging step toward hydrogen production to mitigate fossil fuel dependence. In nature, water oxidation is catalyzed by the Mn_4_CaO_*x*_ cluster in photosystem-II, but the design of synthetic molecular catalysts still remains a challenge. A few catalysts with low-cost abundant cobalt metal ions have been previously reported, although with low durability and high overpotentials. Here, we report two cobalt cluster catalysts with very low overpotentials and high stability for electrochemical water splitting. These two highly efficient heterogeneous bifunctional (BF) electrocatalysts (ECs), formulated as [Co_3_L_4_(H_2_O)_2_]·2.5H_2_O (**Co3**) and [Co_4_L_4_Cl_4_] (**Co4**), (L^2–^ = ethyl-2-(picolinoylimino)­propanoate), are readily prepared from economical and nontoxic starting materials. The distortions of the coordination geometry around the cobalt atoms, due to the steric effects of the bulky ligand (L), modify the electronic environment of the cobalt centers and facilitate water coordination and subsequent splitting. Furthermore, targeted molecular level modifications on previously reported clusters have provided insight into multimetallic cooperativity and structure–activity relationships. Interestingly, **Co4**, having a hitherto unknown Co_4_O_4_ core, acts as an efficient water splitting EC. **Co4** shows a higher activity than **Co3** and very low overpotentials (η) for both the oxygen evolution reaction (OER) and hydrogen evolution reaction (HER) at 10 mA cm^–2^ (η = 157 mV for the OER and 39.8 mV for the HER) and small Tafel slopes (40.0 mV dec^–1^ for the OER and 40.4 mV dec^–1^ for the HER). Additionally, **Co4** also shows a high-performance alkaline H_2_O electrolyzing capacity with a cell voltage of 1.486 V at 10 mA cm^–2^ and exhibits remarkable long-term stability. Thus, our cheap BF molecular EC clearly opens up an innovative platform for scalable O_2_ and H_2_ production.

## Graphical abstract

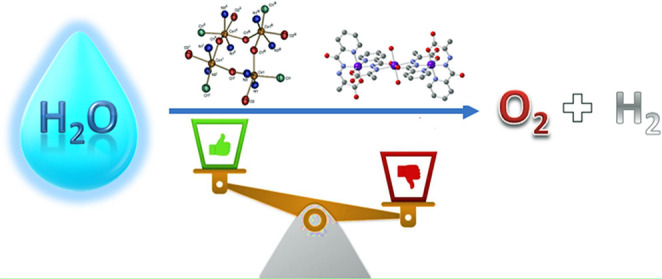

## 1 Introduction

Water splitting has become one of the hottest research areas in Materials Science due to ongoing challenges in the energy and environmental crisis issues.^1–3^ Synthetic molecular electrocatalysts (ECs) for water splitting based on Pt-group metals have limitations owing to their low abundance and consequent high cost together with the concerns about the long-term viability of these catalysts.^4,5^

Great progress has been made over the past years to develop water-splitting catalysts (WSCs) from earth-abundant transition metals.^6–9^ The practical implementation of such WSCs is limited because electrochemical water splitting is a strongly uphill reaction with a large overpotential. Commercial electrolyzers typically operate at cell voltages of 1.8–2.0 V, which is much higher than the theoretical minimum value of 1.23 V.^10^ To overcome this overpotential, synthetic chemists have tried to design efficient nonprecious metal WSCs since the 1980s, when the first non-noble metal cobalt (Co) catalysts for water splitting were described.^11,12^ After the first publications of cobalt polyoxometalates (POMs) as active and stable catalysts for water oxidation, Co-based WSCs have become a very active topic.^13^ Interestingly, a number of cobalt compounds that were initially thought to be water oxidation catalysts (WOCs) were later confirmed as decomposition products of the precursor catalysts, as the true catalytic process is heterogeneous in nature.^14^ Therefore, great care has to be taken to identify if the actual WOC species is the used molecular complex or a decomposition product. The main advantage of molecular WOCs is the capacity to adjust their redox and kinetic properties through molecular engineering on the ligand and/or redox active metal centers. This advantage has been used to prepare some heterogeneous^15^ and homogeneous^16^ molecular WOCs based on cobalt complexes. In some cases, the molecular WSCs are unstable during electrolysis, resulting in the formation of heterogeneous CoO_*x*_.^17^ Thus, compound [(TPA)­Co­(μ–OH)­(μ-O_2_)­Co­(TPA)]^3+^ (TPA = tris­(2-pyridylmethyl)­amine) was initially reported as the first dinuclear Co-based WOC,^18^ but a reinvestigation of this catalytic system revealed that water oxidation most likely is catalyzed by CoO_*x*_ at an overpotential of 550 mV.^19^ In the same way, the cubane cobalt complex [Co^III^_4_O_4_(OAc)_4_(py)_4_] was initially reported as molecular WOC,^20^ but later it was shown that it contains Co^II^ impurities that were actually the source of the active species.^21^ Other studies have pointed out that the Co_4_O_4_ core is better than other hexanuclear cores for WOC.^22^

The use of redox active ligands to overcome the problem of high-valent Co species seems to be one of the key design principles in the development of Co-based WOCs, as it reduces the problem of Co^2+^ leaching or catalyst degradation by formation of CoO_*x*_ species.^23^ Another promising strategy is modification and distortion of the coordination geometry. It has been suggested that the modification of the coordination geometry changes the catalytic activity of different catalysts.^24^ Using this strategy, we have designed two cobalt complexes with a bulky ligand featuring a linear trinuclear metal cluster (**Co3**) or a [2 × 2] grid cluster (**Co4**), both acting as electrocatalysts for water splitting. As far as we know, these are the first trinuclear and tetranuclear cobalt complexes with such catalytic activity. As a WSC, the [2 × 2] grid cluster (**Co4**) gives much better results than the linear trinuclear complex (**Co3**) due to the presence of a Co_4_O_4_ core and more accessible active sites, favoring the adsorption of water-splitting intermediates. Additionally, the larger number of adjacent redox-active metal centers in **Co4** facilitates water activation and ensures intramolecular O–O bond formation. The overall structural and electronic properties of these complexes catalyze the water-splitting process while remaining stable under the reaction conditions, making them extremely promising agents for water oxidation.

## 2 Results

### 2.1 Synthesis of **Co3** and **Co4**

We have synthesized two cobalt complexes formulated as [Co_3_L_4_(H_2_O)_2_]. 2.5H_2_O (**Co3**) and [Co_4_L_4_Cl_4_] (**Co4**) with the redox-active tetradentate ligand ethyl-2-(picolinoylimino)­propanoate (L^2–^). We observe that the change of the precursor Co^II^ salt (Co­(NO_3_)_2_·6H_2_O for **Co3** and CoCl_2_·6H_2_O for **Co4**) results in a change of the nuclearity of the complex (three in **Co3** and four in **Co4**, Figures S1 and S2, respectively). We also observe that the use of a bulky and rigid ligand (H_2_L) results in large distortions of the coordination geometries (especially in **Co4**) that modify the steric and electronic environment around the cobalt centers (see below).

### 2.2 Crystal Structures of **Co3** and **Co4**

Single crystal X-ray analysis revealed that **Co3** crystallizes in the triclinic *P*-1 space group (Table S1). The structure consists of a centrosymmetric trinuclear Co^III^_2_Co^II^ complex (Figure 1a) formulated as [Co_3_(L)_4_(H_2_O)_2_] (L^2–^ = ethyl-2-(picolinoylimino)­propanoate dianion) with two and a half crystallization water molecules. The asymmetric unit contains one Co^II^ center (Co2, located on an inversion center, with an occupancy of 12 ), one Co^III^ center (Co1), two L^2–^ ligands, one coordinated water molecule, and a total of 1.25 water molecules disordered each over two positions with occupancy factors 0.1/0.9, 0.25/0.75 and 0.4/0.6, all located on general positions (Figure S3). Application of the inversion center generates the formula [Co_3_L_4_(H_2_O)_2_]·2.5H_2_O. The X-ray analysis indicates that hydrolysis of this ligand occurred at the terminal ester group, leading to the formation of a carboxylate group (Figure 1a). The main bond distances and angles are displayed in Tables S2 and S3. As expected, the Co1–O and Co1–N bond distances are much shorter (in the range 1.875–1.932 Å) than the Co2–O and Co2–N ones (in the range of 2.047–2.216 Å), confirming the charge assignment of +3 and +2 for Co1 and Co2, respectively and also in agreement with the Bond Valence Sum (BVS) calculations.

**Figure 1:**
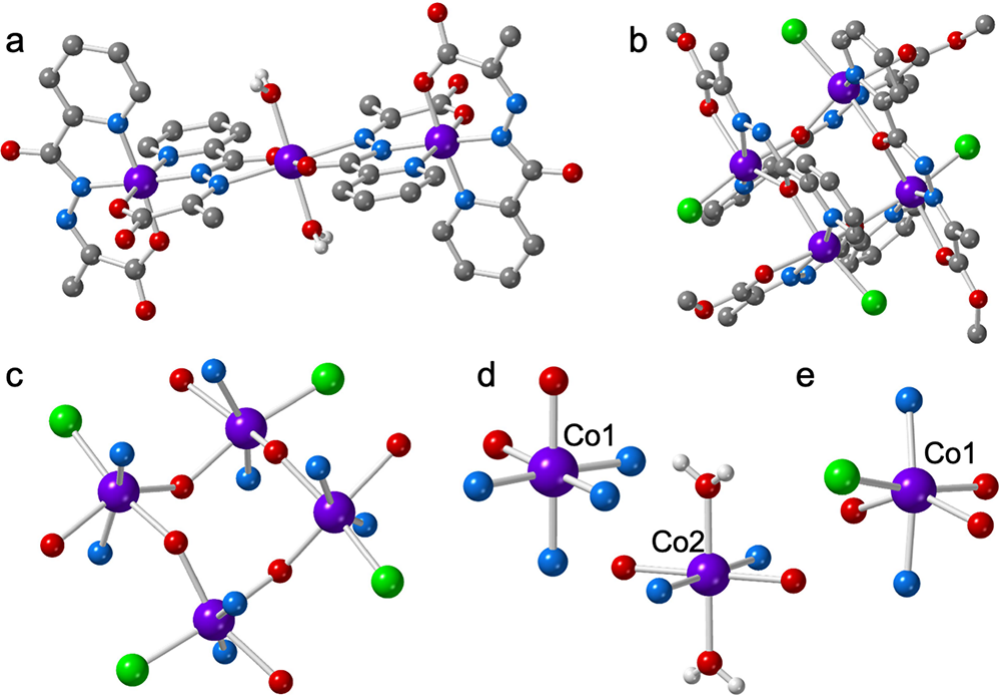
(a) Structure of trimeric complex [Co_3_(L)_4_(H_2_O)_2_] (**Co3**). C-bound H atoms and crystallization water molecules are omitted for clarity. (b) Structure of the tetrameric complex [Co_4_(L)_4_Cl_4_] (**Co4**). H atoms are omitted for clarity, (c) detailed structure of the Co_4_O_4_ cluster in **Co4**, (d) coordination environment of the Co1 and Co2 atoms in compound **Co3**, and (e) coordination environment of the Co1 atom in compound **Co4**. Color code: Co = purple, O = red, C = gray. Cl = green, N = blue, and H = white.

The Co···Co distance between the central (Co2) and terminal (Co1 and Co1^*i*^; *i* = −*x*, −*y*, −*z*) cobalt atoms is 4.8529(6) Å. One of the two independent ligands bridges the central Co2 and terminal Co1 ions acting as a tridentate N_2_O-donor for the terminal cobalt­(III) ions (Co1) and as a bidentate NO-donor for the central cobalt­(II) ion (Co2). The other L^2–^ ligand acts only as a tridentate N_2_O-donor for the terminal cobalt­(III) ions (Co1).

The slightly distorted octahedral N_4_O_2_ coordination about Co1 is provided by four N atoms of the picolinic fragment (N1, N2 and N2, N5) and two carboxylate donor atoms (O3, and O6) of two *mer*-arranged ligands (Figure 1d). The continuous shape (CSh) analysis shows that the coordination geometry around the Co1 atom is very slightly distorted octahedral (CSh = 0.306, Table S5).^25,26^ The best equatorial plane (rms deviation = 0.0348 Å) is formed by the O6, N2, N4, and N5 set of atoms. The Co1–N (mean value of 1.913(9) Å) and Co1–O (mean value of 1.881(5) Å) bond distances are very similar. The *cisoid* and *transoid* angles fall in the range 83.13(13)–94.95(13) and 175.35(13)–176.60(13)°, respectively. The Co1–N2–N3–C7–C9-O3 six-membered chelated ring assumes a flattened twist-boat conformation (puckering parameters: *Q* = 0.186(4) Å, θ = 92.6(11)°, φ = 143.2(11)°), while the Co1–N5–N6–C16–C18-O6 six-membered chelated ring is almost planar (puckering parameters: *Q* = 0.046(4) Å, θ = 63(4)°, φ = −79(6)°). The Co1–N1–C5–C6-N2 and Co1–N4–C14–C15-N5 five-membered chelated rings adopt a flattened envelope conformation (total puckering amplitude of 0.060(3) and 0.077(4) Å, respectively) with the cobalt atoms as the flap.

The Co2 atom exhibits a distorted elongated octahedral coordination geometry of the type N_2_O_4_ with two centrosymmetric pairs of carbonyl atoms (O4, O4^*i*^; *i* = 1 – *x*, −*y*, 1 – z) and water oxygen atoms (O7, O7^i^) forming the equatorial plane and a centrosymmetric pair of imino atoms (N6 and N6^i^) occupying the apical positions (Figure 1d). The CSh analysis (CSh = 2.377, Table S5).^25,26^ shows that the coordination geometry around the Co2 atom is also octahedral and more distorted than that of Co1. The angle formed by the N6–Co2–N6^i^ axis with the normal equatorial plane is 13.41(11)°. The Co2–O7 distance involving the coordinated water molecule (2.074(3) Å) is not remarkably longer than the Co2–O4 distance (2.049) involving the carbonyl O atom. The *cisoid* angles fall in the range 76.67(11)-103.33(11)°, and the *transoid* angles are 180° by symmetry. The Co2–N6–N5–C15-O4 five-membered chelated puckering parameters ring shows an envelope conformation (amplitude *Q* = 0.191(3) Å and φ = −175.2(11)°) with the cobalt atom as a flap. In the crystal, the complex molecules are linked into a three-dimensional network via hydrogen bonds connecting the coordinated and uncoordinated water molecules (Table S4).

Compound [Co_4_L_4_Cl_4_] (**Co4**) crystallizes in the tetragonal *P-*42_1_ *c* space group (Table S1). The asymmetric unit contains a L^2–^ ligand, one Co^II^ ion, and a coordinated Cl atom (Figure S2). The crystal structure of the homoleptic tetranuclear neutral complex **Co4** (Figure S4) contains a **Co**_**4**_ tetrahedrally distorted [2 × 2] square grid of cobalt­(II) ions linked by two sets of approximately parallel ditopic monoanionic ligands arranged orthogonally above and below the [2 × 2] metal pseudoplane (Figure 1b). As can be seen in Figure 1c, the metal ions are bridged by the O1 amide oxygen atom, forming a Co_4_O_4_ arrangement with two oxygen atoms positioned above and two below the least-squares mean plane of the Co_4_ grid (r.m.s. deviation = 0.569 Å). The Co···Co separation between metals bridged by the amide oxygen atom is 3.8933(11) Å. Compound **Co4** results from a head-to-tail arrangement of the ligand strands that leads to the formation of only one type of chromophore, CoN_2_O_3_Cl (Figure 1e), where the Co^II^ atom is surrounded by two nitrogen atoms (belonging to the azomethine group and the pyridine ring from one ligand), three oxygen atoms (two from each of two different amide groups and one from a pyruvate moiety) and one chloride anion (Figure 1e). The continuous shape (CSh) analysis shows that the coordination geometry around the Co1 atom is very distorted octahedral (CSh = 4.532, Table S5).^25,26^ Each ligand interacts with a pair of metal ions in a tetradentate binding mode forming three almost planar five-membered chelated rings (total puckering amplitude of 0.122(4), 0.019(6) and 0.112(4) Å for Co1–O1–C6–N2-N3, Co1–O2–C8–C7-N3 and Co1^i^–O1–C6-C1-N1 respectively; *i* = 1 – *y*, *x*, 1 – *z*). The Co–O_amide_ (mean value 2.13(10) Å), Co–O_carboxylate_ (2.261(4) Å), Co–N_pyridine_ (2.097(4) Å), Co–N_azomethine_ (2.069(3) Å), and Co–Cl (2.3516(16) Å) bond distances fall in the usual range of values reported in the literature. The crystal packing is enforced by weak C–H···Cl hydrogen bonds (Table S4) involving pyridine H atoms. The rigid [2 × 2] grid framework of cobalt ions in the tetranuclear core of **Co4** is expected to serve as the active site for the formation of the O–O bond formation.

### 2.3 Magnetic Properties of **Co3** and **Co4**

The magnetic properties of **Co3** show the expected behavior for an isolated high spin Co^II^ ion with an orbital contribution and spin–orbit coupling. Thus, the product of the molar magnetic susceptibility times the temperature (χ_m_ *T*) shows a value of 3.0 cm^3^ K mol^–1^ at room temperature, close to the expected value for an isolated Co^II^ ion with an orbital contribution, confirming the presence of one Co^II^ and two diamagnetic low spin Co^III^ ions in the trimer.^27^ When the temperature is decreased, χ_m_ *T* shows a progressive decrease and reaches a value of 1.6 cm^3^ K mol^–1^ at 2 K (Figure S5a), indicative of the presence of spin–orbit coupling. Accordingly, we have fit the magnetic data of **Co3** to a simple Co^II^ monomer with an orbital reduction factor (α), a zero field splitting (D) and a spin–orbit coupling (λ) using the program PHI.^28^ This model reproduces very satisfactorily both the magnetic data with the following parameters: α = −1.24(1), λ = −95.6(6) cm^–1^ and |*D*| = 1.52(5) cm^–1^ (solid line in Figure S5a).

The magnetic properties of **Co4** show similar behavior with a χ_m_T value close to 12 cm^3^ K mol^–1^ (i.e., around 3.0 cm^3^ K mol^–1^ per Co^II^ center). When the sample is cooled, the χ_m_ *T* value shows a progressive decrease to reach a value of 4.8 cm^3^ K mol^–1^ at 2 K (Figure S5b). Since **Co4** is a square complex with four identical Co–O–Co bridges, we have fitted the magnetic data with a simple square model with four identical coupling constants (*J*) along the sides of the square, using the program PHI.^28^ We assume that the orbital reduction factor, the zero field splitting, and the spin–orbit coupling are the same for the four Co^II^ centers since they are equivalent. This model reproduces very satisfactorily the magnetic properties with the following parameters: *J* = −1.13(2) cm^–1^, α = −0.92(1), λ = −92(1) cm^–1^ and |*D*| = 3.2(1) cm^–1^ (solid line in Figure S5b, the Hamiltonian we have used is of the type *H* = −*J* [*S*_1_ *S*_2_ + *S*_2_ *S*_3_ + *S*_3_ *S*_4_ + *S*_4_ *S*_1_]).

### 2.4 Electrocatalytic Activity Studies

The electrocatalytic activity of **Co3** and **Co4** for both oxygen and hydrogen evolution reactions (OER and HER) has been investigated through electrochemical impedance spectroscopy (EIS) measurements [at overpotentials (η) of 157 mV for the OER and 40 mV for the HER] (see the Supporting Information and Figure S6a,b). These measurements show that **Co4** exhibits a greater electrochemical active surface area (ECSA) value for both the OER (173.7 m^2^ g^–1^) and HER (238.7 m^2^ g^–1^) as compared to **Co3** (56.2 and 140.0 m^2^ g^–1^, respectively). This result indicates that the surface of **Co4** is highly enriched with active sites that contribute to its enhanced electrocatalytic performances. Interestingly, incorporation of Cl and the increase of the nuclearity in **Co4** with respect to **Co3** result in a noticeable rise of the roughness factor (*R*_f_, Table S6) during both the OER and HER, thus increasing the catalytic activity of the EC. This sharp increase of surface roughness in **Co4** for both the OER (2757.9) and HER (3789.6) compared to **Co3** is expected to facilitate the ion-gas transport, leading to an increase in the electrocatalytic OER/HER performances. Therefore, the larger active site density and R_f_ values seem to be at the origin of the higher activity of **Co4**.

#### 2.4.1 OER Activity Studies

In view of the features of the as-prepared **Co3** and **Co4** catalysts, we have studied the electrocatalytic OER/HER activity by linear sweep voltammetry (LSV). Figure 2a shows that both ECs possess H_2_O oxidation capability with **Co4** exhibiting a better OER performance than **Co3** with a low overpotential (η_10_ = 157 mV, at a current density of 10 mA cm^–2^) (Figure 2b, Table S7). The overpotential at a current density of 20 mA cm^–2^ (η_20_) displays an analogous trend with **Co4** having a 70 mV lower overpotential than **Co3** (Table S7), confirming the better electrocatalytic performance of **Co4.** Moreover, the oxygen evolution current of **Co4** is higher than that of **Co3** and reaches a value of 320.7 mA cm^–2^ at η = 570 mV (Table S7, Figure 2c), indicating an enhanced conductivity and faster electron transport in **Co4**. In order to remove the influence of the mass loading, the activity of both catalysts was examined on the basis of the mass-normalized current density (*j*_m_). As can be seen in Figure 2d, **Co4** requires a lower overpotential than **Co3** to reach a mass-normalized current density of 100 A g^–1^. Obviously, the higher mass activity at 1.48 V for **Co4** than for **Co3** (152 vs 77.8 A g^–1^, Figure 2e, Table S7) agrees well with the lower η_10_ and η_20_ values found in **Co4**. Additionally, we performed Tafel slope analysis to study the OER kinetics. This analysis shows a lower Tafel slope for **Co4** (40 mV dec^–1^ compared to 67.5 mV dec^–1^ for **Co3**, Figure 2f), indicating a better electron transfer rate and faster OER kinetics in **Co4**.

**Figure 2:**
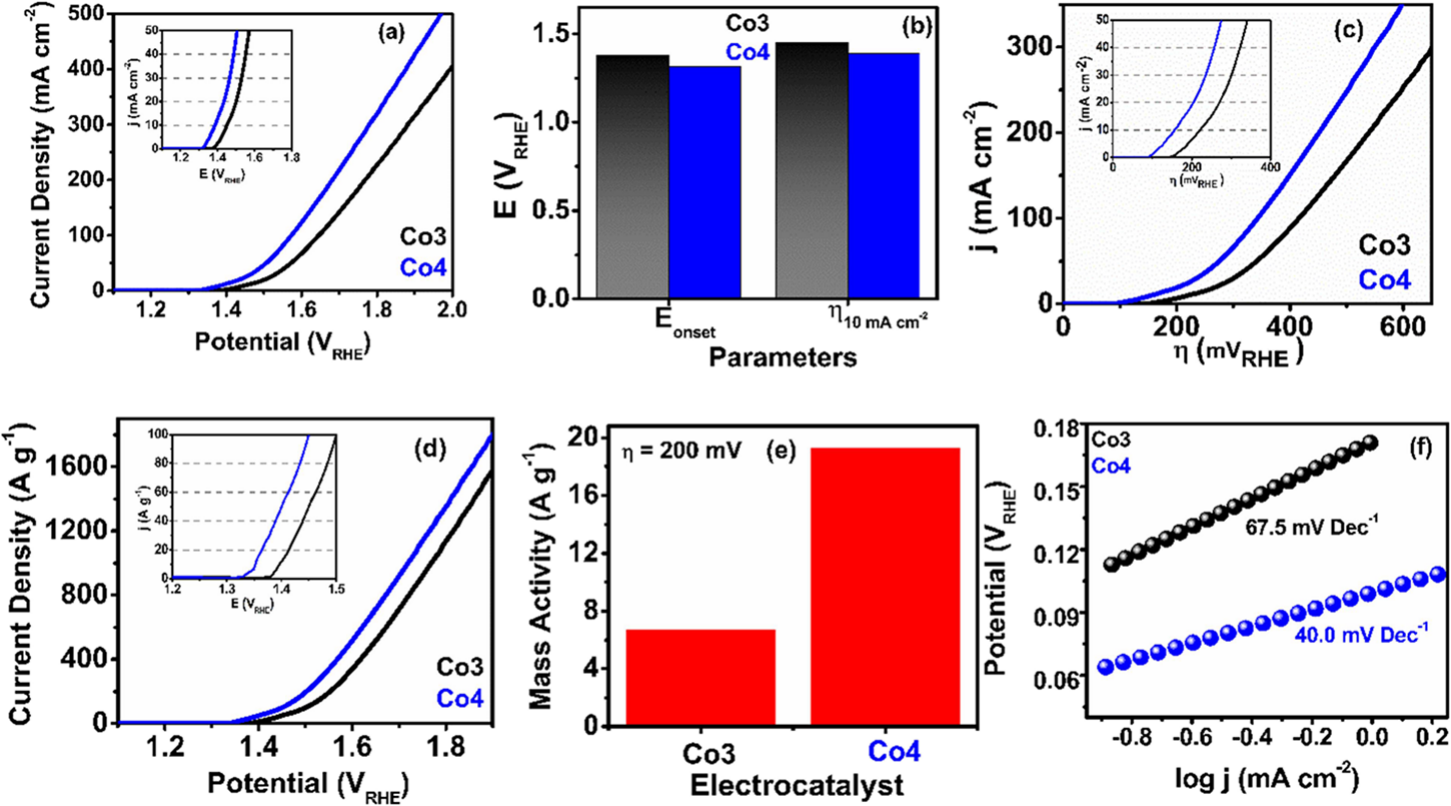
OER activities of the ECs. (a) OER polarization curves in 1 M KOH under 1600 rpm rotational speed (inset, LSV plot up to a current density of 50 mA cm^–2^), (b) bar diagram representing *E*_onset_ and η_10_, (c) the current density vs η plot (inset shows the LSV plot up to a current density of 50 mA cm^–2^), (d) mass activity plots (inset shows the LSV plot up to a current density of 100 A g^–1^), (e) bar diagram representing mass activity at η = 200 mV, and (f) OER Tafel plots of **Co3** and **Co4**.

According to the literature, for **Co3** we propose the OER reaction mechanism shown in Scheme S1. The probable initial state would be the release of a H^+^ from the Co^II^–OH_2_ bond due to its strong acidity. A second deprotonation and electron release from the OH group attached to the Co^III^ center leads to the oxidation of Co^III^ to Co^IV^=O. A H_2_O molecule coordinates then to the Co^IV^=O site in concert with the transfer of a proton to generate a Co^III^–O–O–H site. A deprotonation at this Co^III^–O–O–H facilitates the release of O_2_ followed by the coordination of a water molecule to recover the initial state and close the catalytic cycle.

Also based on the literature, we propose for **Co4** the OER reaction mechanism displayed in Scheme 1. For compound **Co4**, the water oxidation process would start with the removal of 4 electrons in two consecutive steps and the insertion of two hydroxyl groups in two consecutive cobalt centers by breaking the bridging O–O bond, resulting in the formation of two Co^III^–OH groups. As all the Co^III^ centers possess similar ligand environments, deprotonation of one OH group attached to any Co^III^ atom leads to the oxidation of Co^III^ to Co^IV^ in the tetranuclear Co complex which, together with sequential or simultaneous deprotonation, results in the formation of the peroxide species, which is rapidly converted to an O–O bridged intermediate as the close proximity of adjacent metal centers (3.893 Å).^29^ The special geometry of the Co^II^_4_O_4_ core is expected to favor the formation of an O–O bond formation. The O–O bond construction may proceed between cofacial oxido groups as proposed by Masaoka and co-workers until the liberation of O_2_ closes the catalytic cycle.^30^

**Scheme 1:**
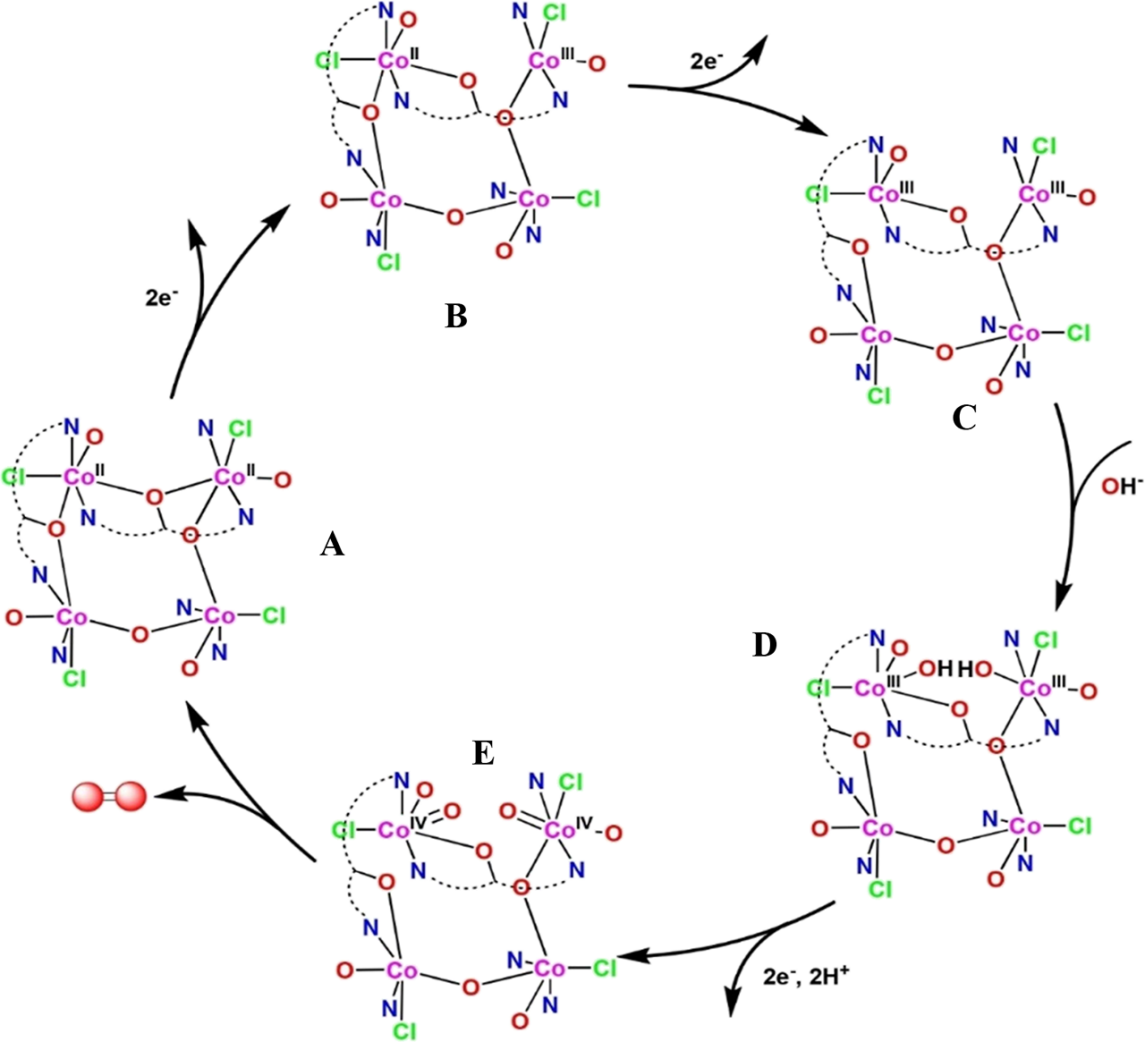
Proposed Catalytic Cycle for the Production of Oxygen Mediated by **Co4**

The Tafel plot shows a Tafel slope of 40.0 mV dec^–1^ for **Co4** indicating that the formation of the surface species is the rate-determining step (RDS).^31^ The higher nuclearity and the presence of Cl ligands would explain the higher OER activity of **Co4** as the catalytic efficiency of complex-based ECs strongly depends on their electronic properties, surface affinity, and steric bulkiness. **Co4** with higher nuclearity provides multiple active sites, which can help to facilitate the OER by providing more pathways for electron transfer and better coordination environments for adsorption and activation of oxygenated reaction intermediates. Moreover, in **Co4**, due to the rigidity of the molecular structure, the rotation of the Co^IV^=O units will be hindered, facilitating the intramolecular oxido–oxido coupling which leads to the formation of face-to-face Co–O–O–Co bridges (because two consecutive Co^IV^=O unit are in *syn-*position), predominantly enhancing the catalytic activity.^32^ In addition, the more electronegative Cl^–^ ligands modify the electronic structure and density of the Co centers and facilitate their oxidation thanks to the inductive effect of the Cl^–^ ligands. This can facilitate charge transfer during the mechanism of the OER mechanism. Furthermore, Cl^–^ ligands can influence the binding strength of intermediates on the catalyst surface, optimizing the adsorption of intermediates which are essential for OER. Thus, the significantly lower onset potential (1.32 V) and η_10_ (0.157 V) observed for **Co4** compared to **Co3** (1.38 and 0.220 V, respectively) can be attributed to the more rigid core structure of **Co4** as well as to the substantial electron-withdrawing ability of the Cl^–^ ligands, rendering **Co4** more hydrophilic than **Co3**.

#### 2.4.2 OER Stability Studies

From a practical point of view, ECs require adequate stability to operate at high current densities for commercial use. Therefore, we have also tested the long-term catalytic durability with chronopotentiometric measurements under a constant current of 10 mA cm^–2^. As can be seen in Figure 3a, **Co4** exhibits a negligible change in η compared to **Co3** even after 30 h of continuous OER test which is further confirmed by their polarization curves after the 5000-cycle stability test (Figure 3b,c).

**Figure 3:**
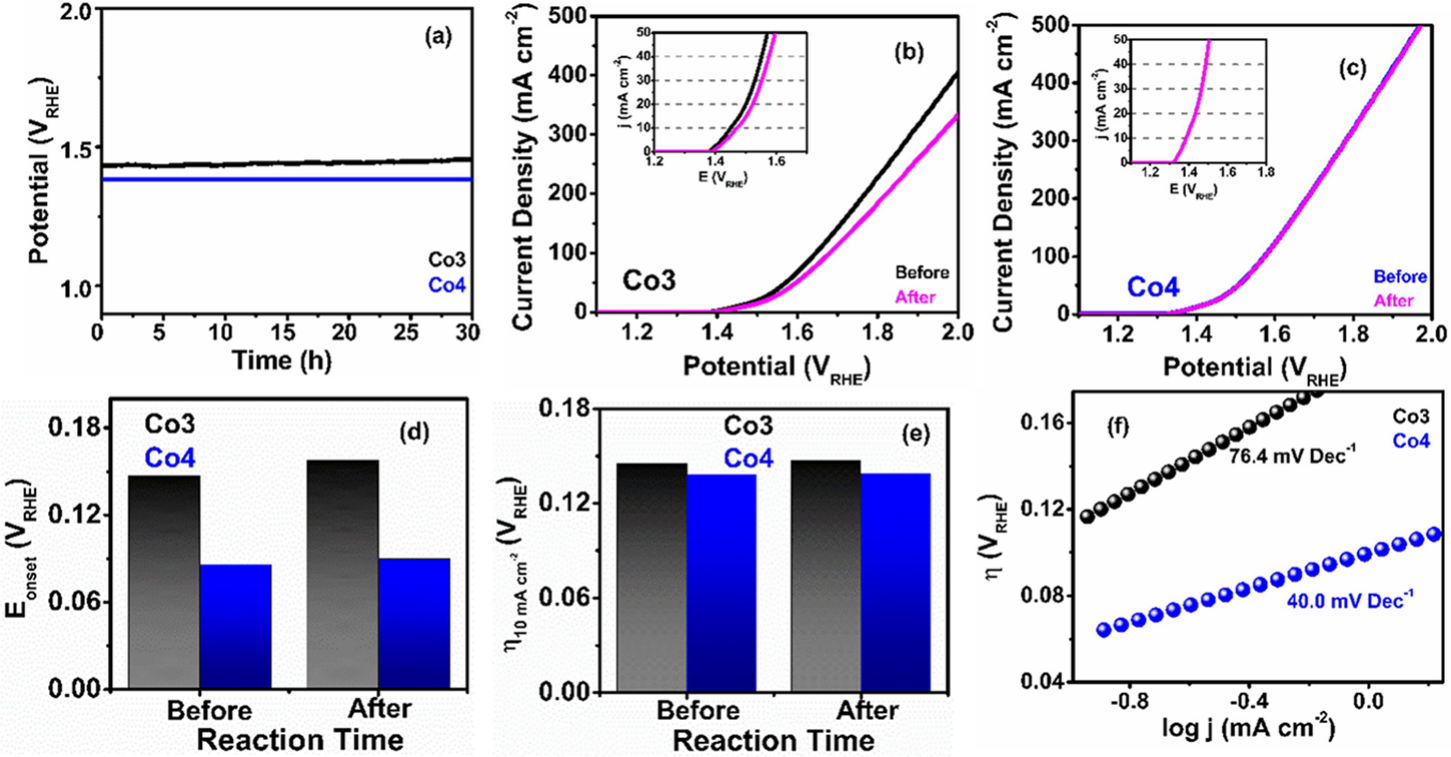
Stability of ECs during the OER. (a) Chronopotentiometric measurements of ECs at 10 mA cm^–2^ for 30 h in 1 M KOH under 1600 rpm rotational speed; OER polarization curves in 1.0 M KOH under 1600 rpm rotational speed of (b) **Co3** and (c) **Co4** before and after the 5000-cycle stability test (insets show the LSV plots up to a current density of 50 mA cm^–2^); bar diagram of (d) *E*_onset_ and (e) η_10_ before and after the 5000-cycle stability test; (f) OER Tafel plots after 30 h stability test.

In **Co4**, no obvious attenuation of the overpotential (η) could be observed in the LSV curves upon 5000 cycles of stability (Table S7), whereas **Co3** experiences a comparatively poorer durability with a very tiny increase of *E*_onset_ and a very small loss of η_10_ (20 mV) (Figure 3d,e). This result further strongly supports the higher activity and robustness of **Co4** over long catalytic reaction periods. This excellent durability of **Co4** is also confirmed by its similar Tafel slope after long-term stability tests, in contrast to **Co3**, whose slope increases from 67.5 to 76.4 mV dec^–1^ (Table S7), reflecting the faster deactivation of **Co3** over time.

#### 2.4.3 HER Activity Studies

In order to facilitate subsequent pairing reactions with the OER process, the HER property of **Co3** and **Co4** is again thoroughly executed in 1.0 M KOH solution. Interestingly, both molecular catalysts possess high HER activity, as shown in Figure 4a. The polarization curve of **Co4** exhibits a fast raise of the cathodic current density with an onset potential, *E*_onset_ = 34.1 mV, lower than that observed for **Co3** (53.3 mV, Table S8, Figure 4b). Moreover, for **Co4**, the overpotentials needed to reach current densities of 10 and 20 mA cm^–2^ (η_10_ and η_20_, respectively) are also lower (39.8 and 64.5 mV, respectively) than those observed for **Co3** (70.2 and 105.3 mV, respectively, Table S8, Figure 4c), suggesting a higher HER efficiency for **Co4**, as also observed for the OER performance.

**Figure 4:**
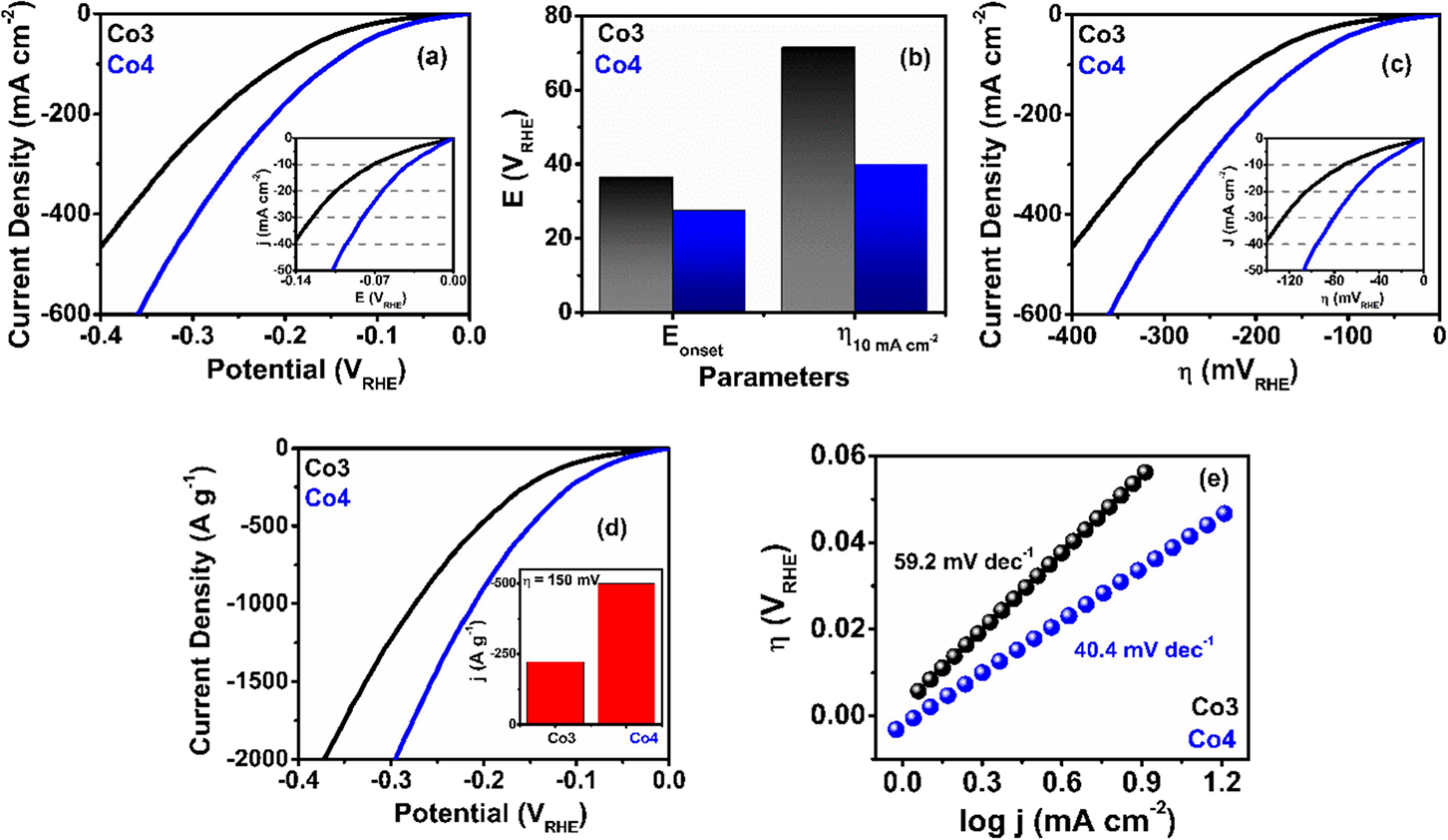
HER activities of the ECs. (a) HER polarization curves in 1.0 M KOH under 1600 rpm rotational speed, (b) bar diagram representing *E*_onset_ and η_10_, (c) LSV_HER_ vs overpotential plot, (d) mass activity plot (inset shows the bar diagram of mass activity at η = 150 mV), and (e) Tafel plots of both ECs.

The mass activity of **Co4** is also found to be higher than that of **Co3** (Figure 4d). Thus, for an overpotential of 150 mV, the mass activity of **Co4** (500 A g^–1^) is more than twice the mass activity of **Co3** (221.2 A g^–1^, Figure 4d and Table S8), further confirming the higher HER activity of **Co4**. Additionally, the smaller Tafel slope for **Co4** (40.4 mV dec^–1^) as compared to **Co3** (59.2 mV dec^–1^, Figure 4e, Table S8) indicates the more facile and faster OER kinetics on **Co4** through efficient electron-mass transportation. The Tafel slope of **Co4** suggests that the HER occurs through the Volmer–Heyrovsky mechanism (eqs S7 and S8) wherein the Heyrovsky reaction is the RDS, the details of which are presented in the Supporting Information.

Under alkaline conditions, the whole reaction rate is influenced by the Volmer step, because an additional H_2_O dissociation step is required. Hence, the Volmer step might be the RDS of the HER. The Volmer process for the alkaline HER should be expressed as two steps: (i) H_2_O → H^·^ + OH^·^ and (ii) OH^·^ + e^–^ → OH^–^. Herein, the experimental results indicate that the electronic state of adjacent Co and O sites synergistically facilitates the subsequent adsorption of OH^·^ and H^·^, respectively, which accelerates the first step of the Volmer process. Then, OH^·^ can easily desorb from the Co site in **Co4**, which facilitates the second step of the Volmer process. The rational structure of the **Co4** cluster thus promotes the Volmer process, leading to excellent HER kinetics. Thus, the lower HER activity of **Co3** compared to **Co4** suggests the fact that an increase of the nuclearity and the presence of Cl^–^ ligands synergistically enhances the HER activity of **Co4**. Moreover, the Tafel slopes suggest that hydrogen production takes place via the Volmer-Heyrovsky mechanism (eqs S7 and S8 in the Supporting Information) rather than via the Tafel one (eq S9) where the electrochemical desorption step is the RDS for the overall HER (eq S10). Based on the literature, we propose the HER reaction pathway shown in Scheme S2.^33^

A further proof of the better performance for the HER of **Co4** is provided by the higher value of the turnover frequency (TOF, eq S15), with values of 1.4 × 10^–2^ s^–1^ for **Co4** vs 0.5 × 10^–2^ s^–1^ for **Co3** at a constant overpotential of 113 mV.

#### 2.4.4 HER Stability Studies

To check the long-term durability, we performed chronopotentiometric analysis on both compounds under a constant current of 10 mA cm^–2^. As can be seen in Figure 5a, an increase in η_10_ occurs for **Co3** whereas **Co4** shows almost no change in η_10_ even after 25 h of the stability test. This result was further confirmed by the polarization curves after 5000 cycles of stability test (Figure 5b,c) that show for **Co3** an increase in η_10_ of 5 mV after the cycle stability tests (Table S8, Figure 5d). The lower durability of **Co3** may be attributed to its partial degradation, which may be due to the peeling off of the compound from the electrode surface during the HER. Additionally, the enhanced activity of **Co4** after the stability test is further confirmed by its slight decrease in the Tafel slope after the stability tests, in contrast to the increase observed in **Co3** (Table S8, Figure 5e).

**Figure 5:**
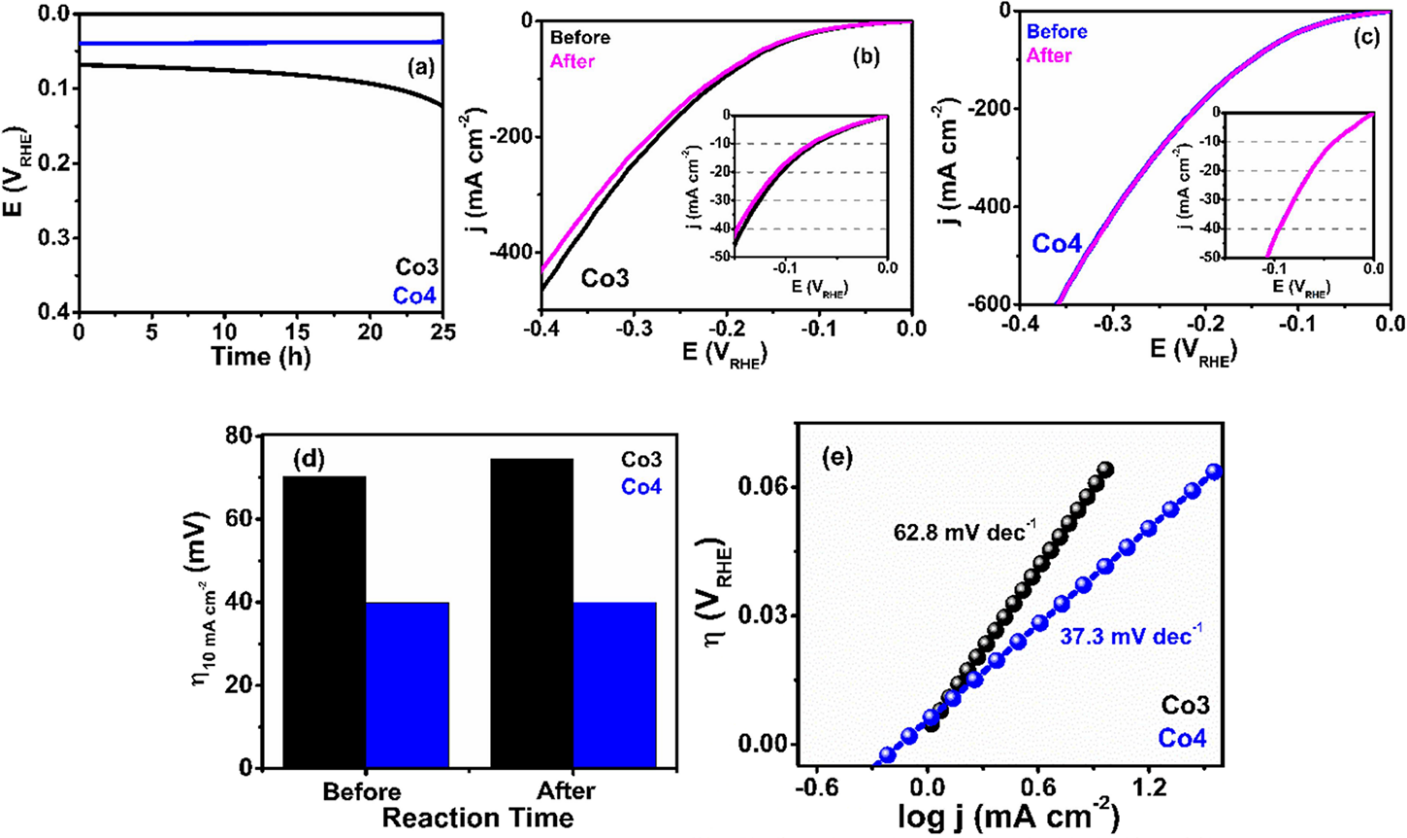
Stability of ECs during the HER. (a) Chronopotentiometric measurements of ECs at 10 mA cm^–2^ for 25 h in 1.0 M KOH under 1600 rpm rotational speed; HER polarization curves in 1.0 M KOH under 1600 rpm rotational speed of (b) **Co3** and (c) **Co4** before and after the 5000-cycle stability tests; (d) bar plot of the η_10_ before and after 5000-cycle stability tests; and (e) HER Tafel plots after 25 h stability test.

This high HER catalytic activity of **Co4** may be attributed to a synergistic effect of different factors: (i) the enhanced charge transport as reflected by the electrochemical impedance spectroscopy (EIS, Figure S6b), (ii) the higher number of active sites, as confirmed by the large accessible electrochemically active surface area (ECSA, Table S6), (iii) the ligand conformational effect, and (iv) a strong interaction between the tetranuclear Co cluster and the Schiff-base ligands. These factors can be attributed to several reasons as follows: (i) the larger number of active cobalt­(II) centers in **Co4** (four centers per complex), (ii) the disposition of the active cobalt­(II) centers in the complex that facilitates the synergistic effect between neighboring centers and lowers the activation energy, and (iii) the electronic effect of the Cl^–^ ligands that reduce the electron density on the cobalt­(II) centers, facilitating the intermediate adsorption during OER and HER, resulting in an excellent bifunctional (BF) water splitting activity.

We can, therefore, conclude that the molecular design of **Co4** creates a strong synergistic effect that decreases the H adsorption energy on the catalyst surface and facilitates HER activity.

#### 2.4.5 Overall Water Splitting (OWS) Activity and Stability of **Co4**

The presence of closely spaced active centers with chelating ligands has been demonstrated to promote the intramolecular cooperative effect between the metal centers and to force coordinated H_2_O molecules to stay in a *syn*-*syn* fashion, thereby promoting the formation of the O–O bond through the coupling of neighboring oxido species, thus reducing the energy barrier for the RDS. The coordinated chloride ligands may also facilitate H-bonding with H_2_O and attract them to the surface of the complex. Moreover, high nuclearity and the presence of Cl ligand electronically activate the Co centers for OH adsorption during the OER. Additionally, the O atoms attached to the Co centers attract the H_2_O molecules closer through H-bonding offering an adequate reactant interface that enhances H_2_O splitting in alkaline medium, thus enormously boosting the HER catalytic behavior of **Co4**.

In view of the exceptional BF OER and HER performances of **Co4** and its catalytic stability, we have constructed a symmetric two-electrode alkaline water electrolyzer using **Co4** as both cathodic and anodic materials, to achieve OWS. Our designed electrocatalyst required an overall potential of 1.486 V (Figure 6a) to attain a water-splitting current density of 10 mA cm^–2^, which is even lower (41 mV) than that of standard Pt/C//RuO_2_ (1.527 V).^34^ This result demonstrates that our **Co4** molecular catalysts can be used for electrocatalytic OWS without the need for expensive and very scarce platinum-group metals electrodes.

**Figure 6:**
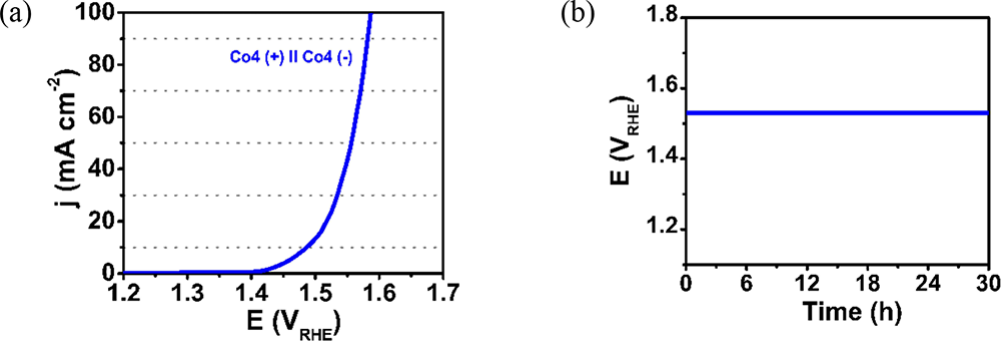
OWS activity and stability of **Co4**. (a) LSV curves of self-assembled alkaline electrolyzers for H_2_O electrolysis, and (b) 30 h chronopotentiometric stability test of the **Co4-**based electrolyzer at a constant current density of 10 mA cm^–2^.

To test the stability of our molecular catalyst, the **Co4**||**Co4** electrolyzer is subjected to chronopotentiometric stability tests for 30 h at a constant current density of 10 mA cm^–2^ (Figure 6b). This measurement shows outstanding stability without any degradation of η_10_ after 30 h, indicative of the robustness of our device. This high stability, together with the high cathodic current density of 100 mA cm^–2^ at an overpotential of 356 mV, entitles our **Co4** complex as a very promising catalyst for electrochemical water splitting devices.

#### 2.4.6 Structural Stability during the Catalytic Cycles of **Co3** and **Co4**

To confirm the structural stability of **Co3** and **Co4**, we have performed Raman analyses (Figure S8), X-ray powder diffraction (XRPD) analysis (Figure S9), scanning electron microscopy (SEM) analysis (Figures S10 and S11), XPS analysis (Figures S12 and S13), and EIS analysis (Figure S14) before and after the catalytic cycles. The Raman spectra revealed characteristic peaks consistent with the original catalyst structure, which clearly indicates the preservation of the molecular arrangements in both compounds. Moreover, XRPD analysis indicates that the crystalline integrity is well-maintained to a large extent. SEM images show a slight modification in the morphology of the catalysts, but the overall structure remains intact. Furthermore, XPS analysis reveals that the oxidation states of the metal centers remain identical to those observed in the single-crystal XRD structure. Also, the absence of any additional peaks supports us to conclude that the catalysts do not undergo significant decomposition during drop casting and catalytic activity. The XPS results combined with the Raman and XRPD analyses indicate that the catalysts retain their structural and chemical integrity under operational conditions, directly supporting the catalytic mechanism. Preservation of the oxidation states, chemical environment, metal–ligand framework, and coordination geometries validate the mechanistic claims. In summary, all of these measurements show no significant changes before and after the catalytic cycles, confirming the stability and robustness of both ECs. Furthermore, these results rule out the possible formation of CoO_*x*_ as well as the loss of the ligands during the electrolysis, further confirming the stability of both ECs.

### 2.5 Discussion

The unique electrocatalytic activity of these two ECs might be related to their redox properties and coordination environments around the Co^II^ centers. Our ECs offer highly available catalytic active sites that assist the selective adsorption of the oxygenated intermediates to a particular site. Thus, on one side, the good permeability of the ions through the channels of the mesoporous structure results in a faster mass transportation and a large surface availability for the OH^–^ ions. On the other side, the redox properties of the cobalt ions lead to a favorable and rapid charge transportation during the catalytic cycle, thanks to a low activation energy and to the easy change in the oxidation states of cobalt from II to III and IV during the OER.

The higher electrocatalytic BF performance of **Co4** compared to **Co3** can be explained by several reasons: (i) A lower charge transfer resistance (*R*_ct_) in **Co4** as shown by electrochemical impedance spectroscopy measurement (Figure S6). This lower resistance leads to a faster electron transfer in **Co4** and thus, an improved catalytic activity; (ii) The greater double layer capacitance (*C*_dl_) (Table S6) of **Co4** during both OER and HER contributes to its largest effective electrochemically active surface area (ECSA) that can offer abundant active sites for boosting its BF activity; (iii) A higher Turn Over Frequency (TOF) value of **Co4** for OER (0.53 s^–1^ at 240 mV) and HER (1.4 × 10^–2^ s^–1^ at 113 mV) compared to **Co3** for OER (0.18 s^–1^ at 240 mV) and HER (0.5 × 10^–2^ s^–1^ at 113 mV). This higher TOF of **Co4** strongly suggests the availability of more exposed and enriched active sites in **Co4** than in **Co3**; (iv) *The ligand effect observed in* **Co4** *.* The higher activity of **Co4** may be also explained by the inductive effect of the coordinated chloride anions that facilitates the electron transfer at the cobalt centers; (v) *The structural conformation in* **Co4**. The disposition of the cobalt ions in the central square Co_4_O_4_ complex in **Co4** enhances the catalytic activity because the [2 × 2] grid structure allows an effective electron transfer and cooperative interactions between the Co^II^ centers, which can facilitate the overall redox processes involved in the OER. Furthermore, the much more distorted coordination geometry of the cobalt­(II) ions in **Co4** (imposed by the rigid skeleton of the ligand) allows the entrance and release of different species during the catalytic cycle. Therefore, the structural conformation of the ligand synergistically enhances both the OER and HER performance of **Co4** compared to **Co3**; (vi) *The composition of the catalyst.* Among the earth abundant transition-metal-based ECs, the cobalt-based ones represent the most appealing candidates to replace the platinum-group metals due to the electronic and redox properties of cobalt ions. In **Co4,** there are four cobalt­(II) ions (only one in **Co3**) that can transfer electrons easily and, together with their close disposition, constitutes a clear advantage when compared with the **Co3** compound.

Finally, in order to compare the OER, HER, and OWS activities of **Co3** and **Co4** with the state-of-the-art catalysts, Tables 1–3 compare the performance of the best reported catalysts for these three reactions (OER, HER, and OWS) with those of compounds **Co3** and **Co4**.

**Table 1:** Comparative Table of the Electrocatalytic OER with Reported State-of-the-Art in 1.0 M KOH

ECs	η (mV)^a^	catalyst loading(mg cm^–2^)	normalized η^a^ with mass loading	Tafel slope (mV dec^–1^)	ref
**Co4**	157	0.20	31.4	40	tw^b^
**Co3**	220	0.20	44.0	67.5	tw^b^
[Fe_2_L_2_(H_2_O)_2_Cl_2_]	190	0.24	45.6	153.5	^24^
[Fe_2_L_2_(H_2_O)_2_(SO_4_)]·2(CH_4_O)	140	0.24	33.6	120.9	
Co_3_(OH)_2_(HPO_4_)_2_	238	2.0	476	69	^35^
CoCo LDH NS	353	0.07	24.71	45	^36^
LDH FeCo	331	0.21	69.51	85	^37^
G-FeCo	277		58.17	60	
SC CoO NRs	330	0.19	62.7	44	^38^
NiCo-hydroxide	208	0.0007	0.14		^39^
CoFe LDH	404	0.1	40.4		^40^
Aza-CMP-Co	289	0.4	115.6	44	^41^
Zn_0.2_Co_0.8_OOH	241	0.204	49.164	35.7	^42^
NiCo-UMOFNs	250	0.2	50	42	^43^
Co^II^TP[Co^III^C]_2_	412	1.0	412	63.6	^44^
Co_3_Mo_3_N/Co_4_N/Co	220			65	^45^
Co–P–B	410	1.4	574	83	^46^
MOF CoTe_2_	220			53.9	^47^
Co_2_(μ–OH)_2_(bbta)	387	0.18	69.66	60	^48^
[Co(C_12_H_6_O_4_(H_2_O)_4_]	520	0.2	104	142	^49^
NiCoFe-HO@NiCo-LDH YSMRs	278	0.33	91.7	49.68	^50^

a *j* = 10 mA cm^–2^.

b tw = this work.

As can be seen in Table 1, compound **Co4** presents the second lowest reported overpotential for the OER with a current density of 10 mA cm^–2^ (157 mV) whereas **Co3** presents the fourth lowest value (220 mV). Moreover, the Tafel slope of **Co4** is the lowest among the reported ECs, demonstrating the superiority of our designed molecular complex in electrocatalysis. Similar types of activity trends are also observed in Tables 2 and 3 for the HER and the OWS reactions, respectively.

**Table 2:** Comparative State-of-the-Art Electrocatalytic HER Activity in 1.0 M KOH

ECs	η (mV)^a^	catalyst loading (mg cm^–2^)	normalized η with mass loading	Tafel slope (mV dec^–1^)	ref
**Co4**	70.2	∼ 0.20	14.0	59.2	tw^b^
**Co3**	39.8	∼ 0.20	8.0	40.4	tw^b^
[Fe_2_L_2_(H_2_O)_2_Cl_2_]	74.1	0.24	17.8	62.6	^24^
[Fe_2_L_2_(H_2_O)_2_(SO_4_)].2CH_4_O	62.0		14.9	45.8	
Co–P–B	270	1.4	378	78	^46^
NW-MnCo_2_O_4_/GDY/CC	150			384	^51^
Co_1_/PCN	89	0.5	44.5	52	^52^
Co@NPCL	87	0.5	43.5	117	^53^
S-CoO NRs	73	0.4	29.2	82	^54^
Co-NG	30	0.285	8.55	82	^55^
Co_2_P/CoP@N–C	161			94	^56^
CuCo-CAT/CC	52	0.408	21.22	51.8	^57^
Co-NC/CF	157	1.1	172.7		^58^
Co50–Mo_2_C-12	125	0.392	49	62	^59^
CoP HoMSs	93	0.5	46.5	70	^60^
CoFeO@BP	88	0.36	31.68	51	^61^
CoMoNiS-NF-31	113	1.86	210.18	85	^62^
Co_3_O_4_/MoS_2_	205	2.0	410	98	^63^
Co_2_Ni_1_N	102	0.24	24.48	60.17	^64^

a *j* = 10 mA cm^–2^.

b tw = this work.

**Table 3:** Comparative State-of-the-Art Bifunctional overall Water Splitting (OWS) Activity in 1.0 M KOH

ECs	catalyst support	*E* (V)^a^ ^,^ ^b^	ref
**Co4/Co4**	Ni foam	1.48	tw^c^
[Fe_2_L_2_(H_2_O)_2_(SO_4_)]·2CH_4_O	Ni foam	1.54	^24^
Ni–Co@Fe–Co PBA	Ni foam	1.60	^65^
Co@NPCL		1.58	^53^
Fe_0.4_Co_0.3_Ni_0.3–1.8_	alloy plate	1.62	^66^
POM@ZnCoS/NF	Ni foam	1.56	^67^
CoP NS	Ni foam	1.63	^68^
CoOx@NC	Ni foam	1.63	^69^
Co_3_(OH)_2_(HPO_4_)_2_	Ni foam	1.95	^35^
(Co/Mo)O_*x*_–Cu	Ni foam	1.86	^70^
CdS@Co_9_S_8_/Ni_3_S_2_	Co/Ni alloy foam	1.56	^71^
Co–Co_0.85_Se	carbon cloth	1.47	^72^
Co_0.59_Fe_0.41_–pydc	Ni foam	1.687	^73^
Co/NBC-900	Ni foam	1.68	^74^
MOF-driven CoS_2_	Ni foam	1.65	^75^
CoS@NiFe LDH	Ni foam	1.65	^76^
CoMoNiS-NF-31	Ni foam	1.54	^62^
FeCo-S/Ni_2_P	Ni foam	1.57	^77^
RhPx/CoNiP_4_O_12_	carbon cloth	1.57	^78^

a vs RHE.

b *j* = 10 mA cm^–2^.

c tw = this work.

The low overpotential values observed for **Co4** for both the OER and HER are further confirmed by the water splitting potential of the **Co4**||**Co4** electrolyzer. Thus, as can be seen in Table 3, **Co4** shows the lowest potential (1.486 V) to attain a water splitting current density of 10 mA cm^–2^ which is below the state-of-the-art potential reported for ECs (Table 3). In summary, the high catalytic activity, low overpotentials, and excellent long-term stability make **Co4** a prospective candidate for future energy applications.

## 3 Conclusions

In conclusion, we have prepared a trinuclear Co_2_^III^Co^II^ complex and a tetranuclear Co^II^_4_ [2 × 2] grid complex that act as very efficient heterogeneous water oxidation catalyst at very low overpotential (specially the **Co4** complex). To improve OER/HER performances of previously reported iron complexes,^24^ we have strategically replace Fe by Co-based electrocatalysts and shown that an increase of the nuclearity and a rigid architecture not only provide stability but also assist the catalytic process by facilitating the O–O bond formation. The ligand used also contributes to the electrocatalytic activity and stability. These results in molecular catalysts may open a new way for the design of novel cobalt complexes with rigid ligands that may act as water splitting catalysts with a low overpotential.

## Supplementary Material



## Data Availability

CCDC Deposition numbers 2325183 (for **Co3**) and 2325184 (for **Co4**) contain the supplementary crystallographic data for this paper. These data can be obtained free of charge from The Cambridge Crystallographic Data Centre via www.ccdc.cam.ac.uk/data_request/cif.

## Notes

Materials and methods; single crystal X-ray structure determination; electrochemical measurements; spectroscopic characterization (PDF)

## References

[1] Berardi S., Drouet S., Francàs L., Gimbert-Suriñach C., Guttentag M., Richmond C., Stoll T., Llobet A. (2014). Molecular
Artificial Photosynthesis. Chem. Soc. Rev..

[2] Dey S., Mondal B., Chatterjee S., Rana A., Amanullah S., Dey A. (2017). Molecular Electrocatalysts
for the Oxygen Reduction Reaction. Nat. Rev.
Chem..

[3] Gil-Sepulcre M., Llobet A. (2022). Molecular Water Oxidation
Catalysts Based on First-Row
Transition Metal Complexes. Nat. Catal..

[4] Choudhury D., Das R., Maurya R., Kumawat H., Neergat M. (2023). Kinetics of the Oxygen
Evolution Reaction (OER) on Amorphous and Crystalline Iridium Oxide
Surfaces in Acidic Medium. Langmuir.

[5] Lee Y., Suntivich J., May K. J., Perry E. E., Shao-Horn Y. (2012). Synthesis
and Activities of Rutile IrO_2_ and RuO_2_ Nanoparticles
for Oxygen Evolution in Acid and Alkaline Solutions. J. Phys. Chem. Lett..

[6] Maayan G., Gluz N., Christou G. (2018). A Bioinspired
Soluble Manganese Cluster
as a Water Oxidation Electrocatalyst with Low Overpotential. Nat. Catal..

[7] Matheu R., Garrido-Barros P., Gil-Sepulcre M., Ertem M. Z., Sala X., Gimbert-Suriñach C., Llobet A. (2019). The Development of
Molecular Water Oxidation Catalysts. Nat. Rev.
Chem..

[8] Kärkäs M. D., Åkermark B. (2016). Water Oxidation
Using Earth-Abundant Transition Metal
Catalysts: Opportunities and Challenges. Dalton
Trans..

[9] Li J., Triana C. A., Wan W., Adiyeri Saseendran D. P., Zhao Y., Balaghi S. E., Heidari S., Patzke G. R. (2021). Molecular
and Heterogeneous Water Oxidation Catalysts: Recent Progress and Joint
Perspectives. Chem. Soc. Rev..

[10] Wang J., Cui W., Liu Q., Xing Z., Asiri A. M., Sun X. (2016). Recent Progress
in Cobalt-Based Heterogeneous Catalysts for Electrochemical Water
Splitting. Adv. Mater..

[11] Brunschwig B. S., Chou M. H., Creutz C., Ghosh P., Sutin N. (1983). Mechanisms
of Water Oxidation to Oxygen: Cobalt­(IV) as an Intermediate in the
Aquocobalt­(II)-Catalyzed Reaction. J. Am. Chem.
Soc..

[12] Elizarova G. L., Matvienko L. G., Lozhkina N. V., Maizlish V. E., Parmon V. N. (1981). Homogeneous
Catalysts for Dioxygen Evolution from Water. Oxidation of Water by
Trisbipyridylruthenium­(III) in the Presence of Metallophthalocyanines. React. Kinet. Catal. Lett..

[13] Stracke J. J., Finke R. G. (2011). Electrocatalytic
Water Oxidation Beginning with the
Cobalt Polyoxometalate [Co_4_(H_2_O)_2_(PW_9_O_34_)_2_]^10‑^:
Identification of Heterogeneous CoO_
*x*
_ as
the Dominant Catalyst. J. Am. Chem. Soc..

[14] Artero V., Fontecave M. (2013). Solar Fuels
Generation and Molecular Systems: Is It
Homogeneous or Heterogeneous Catalysis?. Chem.
Soc. Rev..

[15] Manna P., Debgupta J., Bose S., Das S. K. (2016). A Mononuclear Co ^II^ Coordination Complex Locked in a Confined Space and Acting
as an Electrochemical Water-Oxidation Catalyst: A “Ship-in-a-Bottle”
Approach. Angew. Chem., Int. Ed..

[16] Wasylenko D. J., Ganesamoorthy C., Borau-Garcia J., Berlinguette C. P. (2011). Electrochemical
Evidence for Catalytic Water Oxidation Mediated by a High-Valent Cobalt
Complex. Chem. Commun..

[17] McMillion N. D., Wilson A. W., Goetz M. K., Chang M.-C., Lin C.-C., Feng W.-J., McCrory C. C. L., Anderson J. S. (2019). Imidazole for Pyridine
Substitution Leads to Enhanced Activity Under Milder Conditions in
Cobalt Water Oxidation Electrocatalysis. Inorg.
Chem..

[18] Wang H., Mijangos E., Ott S., Thapper A. (2014). Water Oxidation
Catalyzed
by a Dinuclear Cobalt–Polypyridine Complex. Angew. Chem., Int. Ed..

[19] Wang J.-W., Sahoo P., Lu T.-B. (2016). Reinvestigation
of Water Oxidation
Catalyzed by a Dinuclear Cobalt Polypyridine Complex: Identification
of CoO_
*x*
_ as a Real Heterogeneous Catalyst. ACS Catal..

[20] McCool N. S., Robinson D. M., Sheats J. E., Dismukes G. C. (2011). A Co_4_O_4_ “Cubane”
Water Oxidation Catalyst Inspired
by Photosynthesis. J. Am. Chem. Soc..

[21] Ullman A.
M., Liu Y., Huynh M., Bediako D. K., Wang H., Anderson B. L., Powers D. C., Breen J. J., Abruña H. D., Nocera D. G. (2014). Water Oxidation Catalysis by Co­(II) Impurities in Co­(III)_4_O_4_ Cubanes. J. Am. Chem.
Soc..

[22] Smith P. F., Hunt L., Laursen A. B., Sagar V., Kaushik S., Calvinho K. U. D., Marotta G., Mosconi E., De Angelis F., Dismukes G. C. (2015). Water Oxidation by the [Co_4_O_4_(OAc)_4_(Py)_4_]^+^ Cubium Is Initiated
by OH^–^ Addition. J. Am. Chem.
Soc..

[23] Mondal B., Chattopadhyay S., Dey S., Mahammed A., Mittra K., Rana A., Gross Z., Dey A. (2020). Elucidation of Factors
That Govern the 2e^–^/2H^+^ vs 4e^–^/4H^+^ Selectivity of Water Oxidation by a Cobalt Corrole. J. Am. Chem. Soc..

[24] Chatterjee A., Mondal P., Chakraborty P., Kumar B., Mandal S., Rizzoli C., Saha R., Adhikary B., Dey S. K. (2023). Strategic
Synthesis of Heptacoordinated Fe ^III^ Bifunctional Complexes
for Efficient Water Electrolysis. Angew. Chem.
Int. Ed..

[25] Alvarez S. (2015). Distortion
Pathways of Transition Metal Coordination Polyhedra Induced by Chelating
Topology. Chem. Rev..

[26] Alvarez S., Avnir D., Llunell M., Pinsky M. (2002). Continuous Symmetry
Maps and Shape Classification. The Case of Six-Coordinated Metal compoundsElectronic
Supplementary Information (ESI) Available: Tables of CSD Refcodes,
Structural Parameters and Symmetry Measures for the Studied Compounds.
See Http://Www.Rsc.Org/Suppdata/Nj/B2/B202096n/. New J. Chem..

[27] Lloret F., Julve M., Cano J., Ruiz-García R., Pardo E. (2008). Magnetic Properties of Six-Coordinated High-Spin Cobalt­(II) Complexes:
Theoretical Background and Its Application. Inorg. Chim. Acta.

[28] Chilton N. F., Anderson R. P., Turner L. D., Soncini A., Murray K. S. (2013). PHI: A
Powerful New Program for the Analysis of Anisotropic Monomeric and
Exchange-coupled Polynuclear *d* - and *f* -block Complexes. J. Comput. Chem..

[29] Jiang X., Li J., Yang B., Wei X., Dong B., Kao Y., Huang M., Tung C., Wu L. (2018). A Bio-inspired Cu_4_O_4_ Cubane: Effective Molecular
Catalysts for Electrocatalytic
Water Oxidation in Aqueous Solution. Angew.
Chem., Int. Ed..

[30] Okamura M., Kondo M., Kuga R., Kurashige Y., Yanai T., Hayami S., Praneeth V. K. K., Yoshida M., Yoneda K., Kawata S., Masaoka S. (2016). A Pentanuclear
Iron
Catalyst Designed for Water Oxidation. Nature.

[31] Shinagawa T., Garcia-Esparza A. T., Takanabe K. (2015). Insight on Tafel Slopes from a Microkinetic
Analysis of Aqueous Electrocatalysis for Energy Conversion. Sci. Rep..

[32] Zhang M.-T., Chen Z., Kang P., Meyer T. J. (2013). Electrocatalytic
Water Oxidation with a Copper­(II) Polypeptide Complex. J. Am. Chem. Soc..

[33] Ekspong J., Gracia-Espino E., Wågberg T. (2020). Hydrogen Evolution Reaction Activity
of Heterogeneous Materials: A Theoretical Model. J. Phys. Chem. C.

[34] Mondal P., Baitalik S. (2025). Synergistic Influence
of Multivalent Ru^δ+^ on a CeO_
*x*
_ Nanocatalyst for Self-Powered
Efficient Electrochemical Water Splitting. J.
Mater. Chem. A.

[35] Menezes P. W., Panda C., Walter C., Schwarze M., Driess M. (2019). A Cobalt-Based
Amorphous Bifunctional Electrocatalysts for Water-Splitting Evolved
from a Single-Source Lazulite Cobalt Phosphate. Adv. Funct. Mater..

[36] Song F., Hu X. (2014). Exfoliation of Layered Double Hydroxides for Enhanced Oxygen Evolution
Catalysis. Nat. Commun..

[37] Zhang B., Zheng X., Voznyy O., Comin R., Bajdich M., García-Melchor M., Han L., Xu J., Liu M., Zheng L., García
De Arquer F. P., Dinh C. T., Fan F., Yuan M., Yassitepe E., Chen N., Regier T., Liu P., Li Y., De Luna P., Janmohamed A., Xin H. L., Yang H., Vojvodic A., Sargent E. H. (2016). Homogeneously
Dispersed Multimetal Oxygen-Evolving Catalysts. Science.

[38] Ling T., Yan D.-Y., Jiao Y., Wang H., Zheng Y., Zheng X., Mao J., Du X.-W., Hu Z., Jaroniec M., Qiao S.-Z. (2016). Engineering Surface Atomic Structure
of Single-Crystal Cobalt (II) Oxide Nanorods for Superior Electrocatalysis. Nat. Commun..

[39] Kang J., Qiu X., Hu Q., Zhong J., Gao X., Huang R., Wan C., Liu L.-M., Duan X., Guo L. (2021). Valence Oscillation
and Dynamic Active Sites in Monolayer NiCo Hydroxides for Water Oxidation. Nat. Catal..

[40] Dionigi F., Zeng Z., Sinev I., Merzdorf T., Deshpande S., Lopez M. B., Kunze S., Zegkinoglou I., Sarodnik H., Fan D., Bergmann A., Drnec J., Araujo J. F. D., Gliech M., Teschner D., Zhu J., Li W.-X., Greeley J., Cuenya B. R., Strasser P. (2020). In-Situ Structure
and Catalytic Mechanism of NiFe and CoFe Layered Double Hydroxides
during Oxygen Evolution. Nat. Commun..

[41] Yang H., Li F., Zhan S., Liu Y., Li W., Meng Q., Kravchenko A., Liu T., Yang Y., Fang Y., Wang L., Guan J., Furó I., Ahlquist M. S. G., Sun L. (2022). Intramolecular Hydroxyl
Nucleophilic
Attack Pathway by a Polymeric Water Oxidation Catalyst with Single
Cobalt Sites. Nat. Catal..

[42] Huang Z.-F., Song J., Du Y., Xi S., Dou S., Nsanzimana J. M. V., Wang C., Xu Z. J., Wang X. (2019). Chemical and
Structural Origin of Lattice Oxygen Oxidation in Co–Zn Oxyhydroxide
Oxygen Evolution Electrocatalysts. Nat. Energy.

[43] Zhao S., Wang Y., Dong J., He C.-T., Yin H., An P., Zhao K., Zhang X., Gao C., Zhang L., Lv J., Wang J., Zhang J., Khattak A. M., Khan N. A., Wei Z., Zhang J., Liu S., Zhao H., Tang Z. (2016). Ultrathin
Metal–Organic Framework Nanosheets for Electrocatalytic Oxygen
Evolution. Nat. Energy.

[44] Aljabour A., Awada H., Song L., Sun H., Offenthaler S., Yari F., Bechmann M., Scharber M. C., Schöfberger W. (2023). A Bifunctional
Electrocatalyst for OER and ORR Based on a Cobalt­(II) Triazole Pyridine
Bis-[Cobalt­(III) Corrole] Complex. Angew. Chem.,
Int. Ed..

[45] Liu Y., Wang L., Hübner R., Kresse J., Zhang X., Deconinick M., Vaynzof Y., Weidinger I. M., Eychmüller A. (2024). Cobalt-based
Co_3_Mo_3_N/Co_4_N/Co Metallic Heterostructure
as a Highly Active Electrocatalyst
for Alkaline Overall Water Splitting. Angew.
Chem., Int. Ed..

[46] Silviya R., Bhide A., Gupta S., Bhabal R., Mali K. H., Bhagat B. R., Spreitzer M., Dashora A., Patel N., Fernandes R. (2024). Bifunctional Amorphous Transition-Metal Phospho-Boride
Electrocatalysts for Selective Alkaline Seawater Splitting at a Current
Density of 2A cm^–2^. Small
Methods.

[47] Rosyara Y. R., Muthurasu A., Chhetri K., Pathak I., Ko T. H., Lohani P. C., Acharya D., Kim T., Lee D., Kim H. Y. (2024). Highly Porous Metal–Organic Framework Entrapped
by Cobalt Telluride–Manganese Telluride as an Efficient Bifunctional
Electrocatalyst. ACS Appl. Mater. Interfaces.

[48] Lu X.-F., Liao P.-Q., Wang J.-W., Wu J.-X., Chen X.-W., He C.-T., Zhang J.-P., Li G.-R., Chen X.-M. (2016). An Alkaline-Stable,
Metal Hydroxide Mimicking Metal–Organic Framework for Efficient
Electrocatalytic Oxygen Evolution. J. Am. Chem.
Soc..

[49] Ma T. Y., Dai S., Jaroniec M., Qiao S. Z. (2014). Metal–Organic Framework Derived
Hybrid Co_3_O_4_ -Carbon Porous Nanowire Arrays
as Reversible Oxygen Evolution Electrodes. J.
Am. Chem. Soc..

[50] Niu Q., Yang M., Luan D., Li N. W., Yu L., Lou X. W. (2022). Construction of
Ni-Co-Fe Hydr­(Oxy)­oxide@Ni-Co Layered
Double Hydroxide Yolk-Shelled Microrods for Enhanced Oxygen Evolution. Angew. Chem., Int. Ed..

[51] Qi L., Zheng Z., Xing C., Wang Z., Luan X., Xue Y., He F., Li Y. (2022). 1D Nanowire Heterojunction Electrocatalysts
of MnCo_2_O_4_/GDY for Efficient Overall Water Splitting. Adv. Funct. Mater..

[52] Cao L., Luo Q., Liu W., Lin Y., Liu X., Cao Y., Zhang W., Wu Y., Yang J., Yao T., Wei S. (2019). Identification of Single-Atom Active Sites in Carbon-Based Cobalt
Catalysts during Electrocatalytic Hydrogen Evolution. Nat. Catal..

[53] Gu T., Shen J., Sun Z., Li F., Zhi C., Zhu M., Liu J. (2024). Engineering Non-precious Trifunctional Cobalt-Based
Electrocatalysts for Industrial Water Splitting and Ultra-High-Temperature
Flexible Zinc-Air Battery. Small.

[54] Ling T., Yan D.-Y., Wang H., Jiao Y., Hu Z., Zheng Y., Zheng L., Mao J., Liu H., Du X.-W., Jaroniec M., Qiao S.-Z. (2017). Activating Cobalt­(II)
Oxide Nanorods for Efficient Electrocatalysis by Strain Engineering. Nat. Commun..

[55] Fei H., Dong J., Arellano-Jiménez M. J., Ye G., Dong Kim N., Samuel E. L. G., Peng Z., Zhu Z., Qin F., Bao J., Yacaman M. J., Ajayan P. M., Chen D., Tour J. M. (2015). Atomic Cobalt on Nitrogen-Doped Graphene for Hydrogen
Generation. Nat. Commun..

[56] Arunkumar P., Gayathri S., Han J. H. (2022). Impact
of an Incompatible Atomic
Nickel-Incorporated Metal–Organic Framework on Phase Evolution
and Electrocatalytic Activity of Ni-Doped Cobalt Phosphide for the
Hydrogen Evolution Reaction. ACS Appl. Energy
Mater..

[57] Geng B., Yan F., Zhang X., He Y., Zhu C., Chou S., Zhang X., Chen Y. (2021). Conductive CuCo-Based Bimetal Organic
Framework for Efficient Hydrogen Evolution. Adv. Mater..

[58] Huang H., Zhou S., Yu C., Huang H., Zhao J., Dai L., Qiu J. (2020). Rapid and
Energy-Efficient Microwave Pyrolysis for
High-Yield Production of Highly-Active Bifunctional Electrocatalysts
for Water Splitting. Energy Environ. Sci..

[59] Ma Y., Chen M., Geng H., Dong H., Wu P., Li X., Guan G., Wang T. (2020). Synergistically Tuning Electronic
Structure of Porous β-Mo _2_ C Spheres by Co Doping
and Mo-Vacancies Defect Engineering for Optimizing Hydrogen Evolution
Reaction Activity. Adv. Funct. Mater..

[60] Hou P., Li D., Yang N., Wan J., Zhang C., Zhang X., Jiang H., Zhang Q., Gu L., Wang D. (2021). Delicate Control
on the Shell Structure of Hollow Spheres Enables Tunable Mass Transport
in Water Splitting. Angew. Chem., Int. Ed..

[61] Li X., Xiao L., Zhou L., Xu Q., Weng J., Xu J., Liu B. (2020). Adaptive Bifunctional Electrocatalyst of Amorphous
CoFe Oxide @ 2D Black Phosphorus for Overall Water Splitting. Angew. Chem., Int. Ed..

[62] Yang Y., Yao H., Yu Z., Islam S. M., He H., Yuan M., Yue Y., Xu K., Hao W., Sun G., Li H., Ma S., Zapol P., Kanatzidis M. G. (2019). Hierarchical Nanoassembly of MoS_2_/Co_9_S_8_/Ni_3_S_2_/Ni
as a Highly Efficient Electrocatalyst for Overall Water Splitting
in a Wide pH Range. J. Am. Chem. Soc..

[63] Muthurasu A., Maruthapandian V., Kim H. Y. (2019). Metal-Organic Framework Derived Co_3_O_4_/MoS_2_ Heterostructure for Efficient
Bifunctional Electrocatalysts for Oxygen Evolution Reaction and Hydrogen
Evolution Reaction. Appl. Catal. B Environ..

[64] Feng X., Wang H., Bo X., Guo L. (2019). Bimetal–Organic
Framework-Derived Porous Rodlike Cobalt/Nickel Nitride for All-pH
Value Electrochemical Hydrogen Evolution. ACS
Appl. Mater. Interfaces.

[65] Zhang H., Diao J., Ouyang M., Yadegari H., Mao M., Wang M., Henkelman G., Xie F., Riley D. J. (2023). Heterostructured
Core–Shell Ni–Co@Fe–Co Nanoboxes of Prussian
Blue Analogues for Efficient Electrocatalytic Hydrogen Evolution from
Alkaline Seawater. ACS Catal..

[66] Chen Y., Yang L., Li C., Wu Y., Lv X., Wang H., Qu J. (2024). In Situ Hydrothermal Oxidation of
Ternary FeCoNi Alloy Electrode for Overall Water Splitting. Energy Environ. Mater..

[67] Gautam J., Liu Y., Gu J., Ma Z., Zha J., Dahal B., Zhang L., Chishti A. N., Ni L., Diao G., Wei Y. (2021). Fabrication of Polyoxometalate Anchored Zinc Cobalt Sulfide Nanowires
as a Remarkable Bifunctional Electrocatalyst for Overall Water Splitting. Adv. Funct. Mater..

[68] Wu H., Chen Z., Zhang J., Wu F., Xiao F., Du S., He C., Wu Y., Ren Z. (2018). Generalized Synthesis
of Ultrathin Cobalt-Based Nanosheets from Metallophthalocyanine-Modulated
Self-Assemblies for Complementary Water Electrolysis. Small.

[69] Jin H., Wang J., Su D., Wei Z., Pang Z., Wang Y. (2015). In Situ Cobalt–Cobalt Oxide/N-Doped
Carbon Hybrids As Superior
Bifunctional Electrocatalysts for Hydrogen and Oxygen Evolution. J. Am. Chem. Soc..

[70] Tartour A. R., Sanad M. M. S., El-Hallag I. S., Moharram Y. I. (2024). Novel Mixed Heterovalent
(Mo/Co)­Ox-Zerovalent Cu System as Bi-Functional Electrocatalyst for
Overall Water Splitting. Sci. Rep..

[71] Si F., Tang C., Gao Q., Peng F., Zhang S., Fang Y., Yang S. (2020). Bifunctional CdS@Co_9_S_8_/Ni_3_S_2_ Catalyst for Efficient Electrocatalytic
and Photo-Assisted Electrocatalytic Overall Water Splitting. J. Mater. Chem. A.

[72] Yu J., Yu W., Wang Y., Li X., Liu R., Zhang X., Liu H., Zhou W. (2022). Capture and
Recycling of Toxic Selenite Anions by Cobalt-Based
Metal-Organic-Frameworks for Electrocatalytic Overall Water Splitting. Chem. Eng. J..

[73] Hou J.-J., Liu H., Wang T., Tian B.-Q., Yang Y., Zhang X.-M. (2024). Surface
Defect-Engineered Fe Doping in Layered Co-Based Complex as Highly
Efficient Bifunctional Electrocatalysts for Overall Water Splitting. Dalton Trans..

[74] Liu M., Hong Q., Li Q., Du Y., Zhang H., Chen S., Zhou T., Zhang J. (2018). Cobalt Boron
Imidazolate
Framework Derived Cobalt Nanoparticles Encapsulated in B/N Codoped
Nanocarbon as Efficient Bifunctional Electrocatalysts for Overall
Water Splitting. Adv. Funct. Mater..

[75] Ahn I.-K., Joo W., Lee J.-H., Kim H. G., Lee S.-Y., Jung Y., Kim J.-Y., Lee G.-B., Kim M., Joo Y.-C. (2019). Metal-Organic
Framework-Driven Porous Cobalt Disulfide Nanoparticles Fabricated
by Gaseous Sulfurization as Bifunctional Electrocatalysts for Overall
Water Splitting. Sci. Rep..

[76] Lee Y. J., Park S. (2022). Metal–Organic Framework-Derived
Hollow CoS_
*x*
_ Nanoarray Coupled with NiFe
Layered Double Hydroxides as Efficient
Bifunctional Electrocatalyst for Overall Water Splitting. Small.

[77] Chen P., Wu Y., Guo X., Wang M., Yu C., Jiang H., Zhou W., Wu G., Yan J. (2024). Rational Design of
FeCo-S/Ni_2_P/NF Heterojunction as a Robust Electrocatalyst
for Water Splitting. Inorg. Chem..

[78] Guo B., Zhao J., Xu Y., Wen X., Ren X., Huang X., Niu S., Dai Y., Gao R., Xu P., Li S. (2024). Noble Metal Phosphides
Supported on CoNi Metaphosphate
for Efficient Overall Water Splitting. ACS Appl.
Mater. Interfaces.

