# Supplementary material for: Unveiling Synergistic Effectiveness of Strategically Designed Cobalt Clusters for Efficient Water Electrolysis
Source: ACS Catal. 2025 Jan 27;15(3):2472–83. doi: 10.1021/acscatal.4c06466 (PMC12128174; doi:10.1021/acscatal.4c06466)
Supplement: Supplementary file 1 [file cs4c06466_si_001.pdf]

Supporting Information

Unveiling Synergistic Effectiveness of Strategically Designed Cobalt Clusters for Efficient Water Electrolysis

Abhishikta Chatterjee,[a] *Papri Mondal,*[b] *Priyanka Chakraborty,*[a] *Sourav Mandal,*[a] *Corrado Rizzoli,*[c] *Carlos J. Gómez-García,*[d,*] *Bibhutosh Adhikary,*[b] *Dulal Senapati*[e]*, Subrata K. Dey*[a, *]

[a] Department of Chemistry, Sidho-Kanho-Birsha University, Purulia-723104, WB, India, e-mail: skdchem@skbu.ac.in .

[b] Department of Chemistry, Indian Institution of Engineering Science and Technology, Shibpur, Howrah-711103, India

[c] Dipartimento S.C.V.S.A., Università di Parma, Parco Area delleScienze 17/A, Parma I-43124, Italy

[d] Departamento de Química Inorgánica. Universidad de Valencia. C/ Dr. Moliner 50. 46100 Burjasot (Valencia) Spain. e-mail: carlos.gomez@uv.es

[e] Chemical Sciences Division, Saha Institute of Nuclear Physics, 1/AF Bidhannagar, Kolkata 700064, India.

Table of Contents

Section 1: Materials and Methods

Section 2: Single Crystal X-ray Structure Determination

Section 3: Electrochemical Measurements

Section 4: Spectroscopic Characterization

References

Section 1: Materials and Methods

The H_2L ligand, the salts $Co(NO_3)_2{\cdot}6H_2O$ and $CoCl_2{\cdot}6H_2O$, the solvent MeOH and the base NEt_3 were of analytical grade (Sigma-Aldrich) and were used as received.

Synthesis of $Co_3L_4(H_2O)_2]{\cdot}2.5H_2O$ (Co3)

The complex **Co3** was prepared by adding $Co(NO_3)_2{\cdot}6H_2O$ (1 mmol, 0.291 g) to the ligand (1 mmol, 0.219 g) in 20 mL of hot methanol. Triethylamine (1.5 mmol, 209 µL) was added to the mixture, which was stirred at room temperature for 20 minutes. The resulting red solution was filtered and allowed to stand at room temperature. After two days, red crystals of **Co3** suitable for X-ray analysis were collected (Yield ≈ 50 %). Anal. Calcd (%) for **Co3**: C, 40.08; H, 3.45; N, 15.58 Found (%): C, 40.12; H, 3.51; N, 15.77. IR (cm^{-1}): 3427 (ν H_2O), 1660, 1628 (ν CO, CN), 1031 (ν py) (Figure S7).

Synthesis of $[Co_4L_4Cl_4]$ (Co4)

The complex **Co4** was prepared by adding $CoCl_2{\cdot}6H_2O$ (1 mmol, 0.237 g) to the ligand (1 mmol, 0.219 g) in 20 mL of hot methanol. The solution was stirred at room temperature for 1 h. The resulting red solution was filtered and allowed to stand at room temperature. After two days, red crystals of **Co4** suitable for X-ray analysis were collected (Yield ≈ 50 %). Anal. Calcd (%) for **Co4**: C 38.18; H 3.20; N 13.35 Found (%): C, 38.11; H, 3.18; N, 13.23. IR (cm^{-1}) 3400 (ν H_2O), 1630, 1610 (ν CO, CN), 1022 (ν py) (Figure S7).

Chemical and Physical Characterization

Elemental analyses were performed with a Perkin Elmer 2400 elemental analyzer. The crystallinity and stability of these complexes (before and after the catalytic cycles) were investigated through an X-ray diffractometer (Rigaku, Smartlab) (PXRD) using monochromatic Cu K_α radiation (λ = 1.540598 Å). X-ray photoelectron spectroscopic (XPS) studies were performed to identify the chemical states of the surface atoms with an Omicron Multiprobe spectrometer fitted with an EA-125 hemispherical analyzer. Raman spectra (ReniswaIn Via) were measured in the wavenumber range of 500-2800 cm^{-1} using an excitation of 532 nm generated

from a solid-state 50 mW laser. FT-IR spectra were collected with a Perkin-Elmer FT-IR spectrophotometer in KBr pellets in the 4000-400 cm^{-1} wavenumber range.

X-ray Crystallography

Single crystal X-ray intensity data were collected at room temperature on a Bruker D8 VENTURE Super DUO (for **Co3**) and a Bruker D8 QUEST Photon II (for **Co4**) diffractometer equipped with graphite monochromated Mo K_α radiation (λ = 0.71073 Å). Data collection and reduction were carried out using the APEX4 and SAINT packages.[S1] Multi-scan absorption correction was applied to the intensity data using the SADABS software.[S1] The structures were solved by direct methods using SHELXT[S2] and refined by full-matrix least-squares on F^2 on all unique reflections using SHELXL-2019/3.[S3] The non-hydrogen atoms were refined anisotropically and all C-bound hydrogen atoms were placed geometrically and refined using a riding atom approximation, with C-H = 0.93-0.96 Å, and with U_{iso}(H) = 1.2U_{eq}(C) or 1.5U_{eq}(C) for methyl H atoms. A rotating model was used for the methyl groups.

In **Co3**, the hydrogen atoms of the coordinated water molecule (O7) were located in a difference Fourier map and refined with U_{iso}(H) = 1.5U_{eq}(O) by constraining the O-H distances and H···H separations to be 0.83(1) and 1.312(2) Å, respectively. The crystallization water solvent molecules are disordered over two or four positions around symmetry centers and were refined with site occupation factors of 0.5 (O8 and O9) or 0.25 (O10). In the refinement, ISOR and EADP restraints were used for O9 and O10, respectively. The H atoms of the disordered water molecules were placed in chemically sensible positions on the basis of hydrogen bonding and refined as fixed contributors with U_{iso}(H) = 1.5U_{eq}(O). Two outliers (-3, -9, 5 and -4, -8, 5) were omitted in the last cycles of refinement. The final refinement of **Co4** was carried out as a two-component inversion twin, resulting in a 0.09(3) fraction of the inverted component.

All figures were drawn using the ORTEP-3,[S4] SCHAKAL-99[S5] and CrystalMaker X[S6] programs.

Electrochemical Measurements

All the electrochemical experiments were carried out on a CHI7014E electrochemical analyzer (CH Instruments, USA) using a rotating disc electrode (RDE) in a standard three electrode cell configuration at room temperature. The RDE (disk diameter 4 mm), Ag/AgCl electrode (with saturated KCl solution), and a graphite rod were employed as working reference and counter electrodes, respectively. Purified 1.0 M aqueous KOH solution (pH ~11.6) was used in all the experiments as the electrolyte. This electrolyte was purged with high purity N_2 over 30 min before OER and HER measurements. All electrochemical performances were measured under a rotational speed of 1600 rpm. Initially, the RDE was sequentially cleaned with 0.5 and 0.05 μm alumina powder and then washed with H_2O under sonication for 30 min. The working electrode for both OER/HER was prepared as follows: ≈ 5.2 mg of complex electrocatalyst was dispersed for 1 h through ultrasonication into a mixture of 1 mL of H_2O and 40 μL of nafion (0.5 %) to produce a homogeneous ink. Subsequently, 5 μL of the prepared ink was drop-casted on the surface of a pre-polished glassy carbon disk of the rotating disk electrode (RDE, disk diameter 4 mm), resulting in an electrocatalyst loading of 0.20 mg cm^{-2}. Finally, the electrodes were air-dried for 2 h at room temperature before the electrochemical measurements.

Magnetic Measurements

Variable temperature magnetic susceptibility measurements were carried out with a Quantum Design MPMS-XL-5 SQUID magnetometer, in the temperature range 2-300 K, with an applied magnetic field of 0.1 T, on polycrystalline samples of compounds **Co3** and **Co4** (with masses of 8.656 and 11.211 mg, respectively). The susceptibility data were corrected for the sample holder previously measured using the same conditions and for the diamagnetic contribution of the sample, as deduced by using Pascal's constant tables.[S7]

Section 2: Single Crystal X-ray Structure Determination

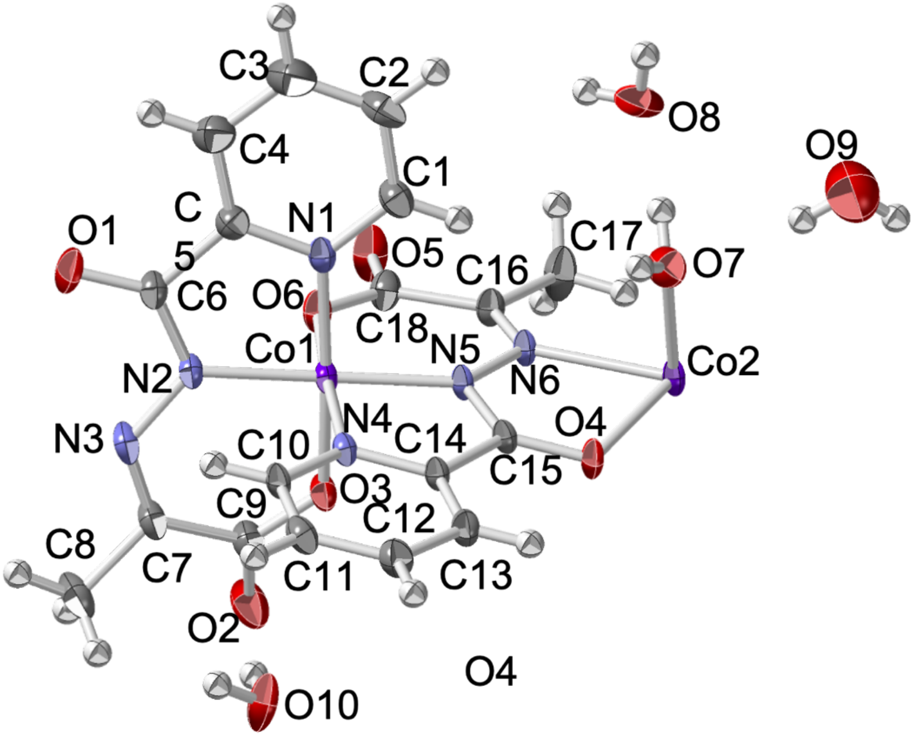

Figure S1. ORTEP drawing of the asymmetric unit of **Co3** with the labelling scheme. Ellipsoids drawn at 30 % probability.

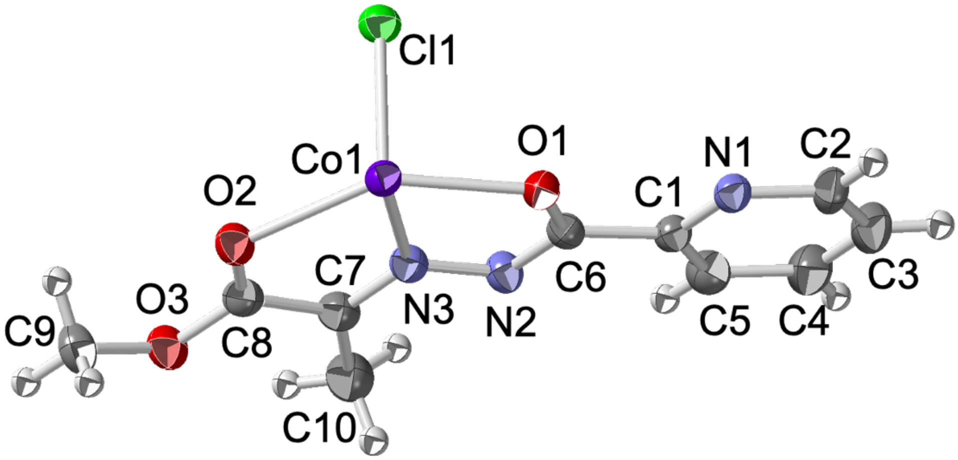

Figure S2. ORTEP drawing of the asymmetric unit of **Co4** with the labelling scheme. Ellipsoids drawn at 30 % probability.

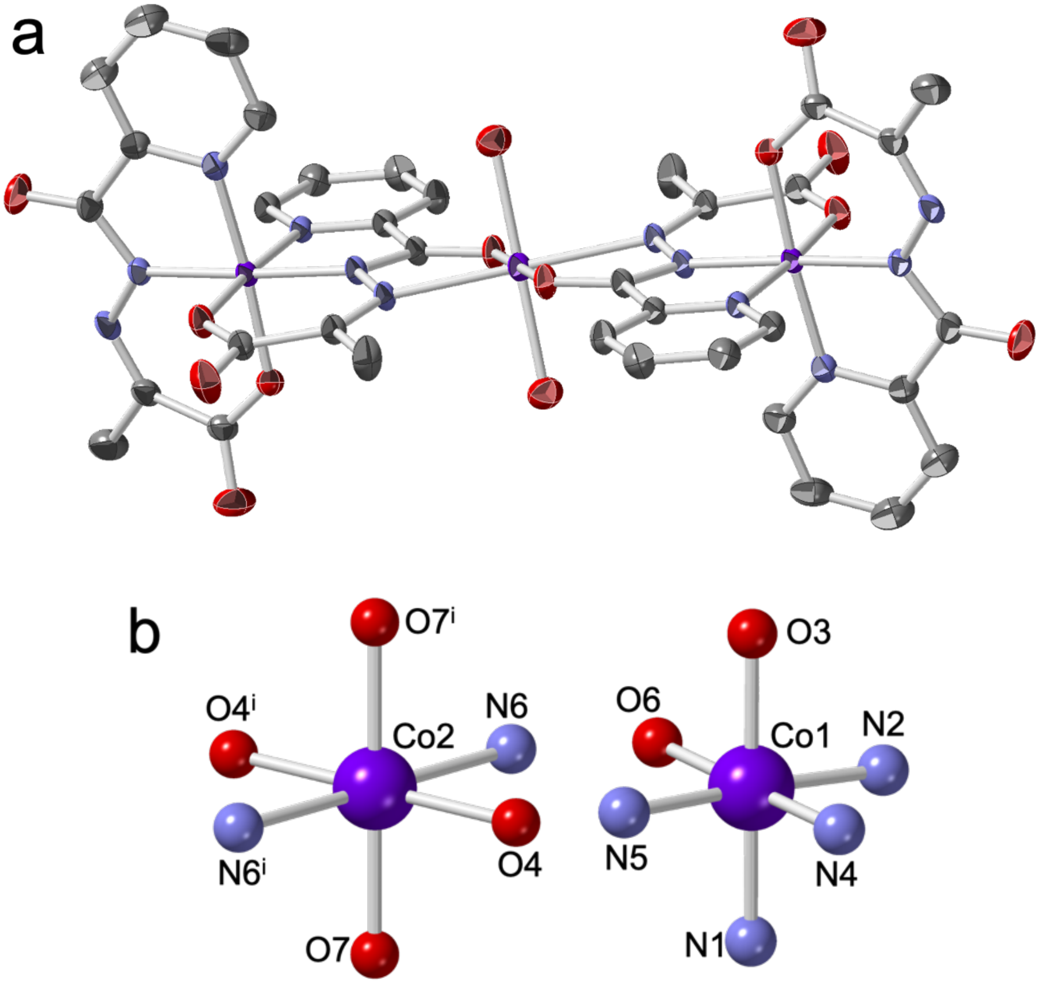

Figure S3. (**a**) ORTEP drawing with the labelling scheme of the structure of the trimeric complex $[Co_3(L)_4(H_2O)_2]$ (**Co3**) (symmetry code: i = -x,-y,-z). Ellipsoids are drawn at 30 % probability level. H atoms and crystallization water molecules are omitted for clarity. (**b**) Coordination environment of the Co1 and Co2 atoms in compound **Co3**.

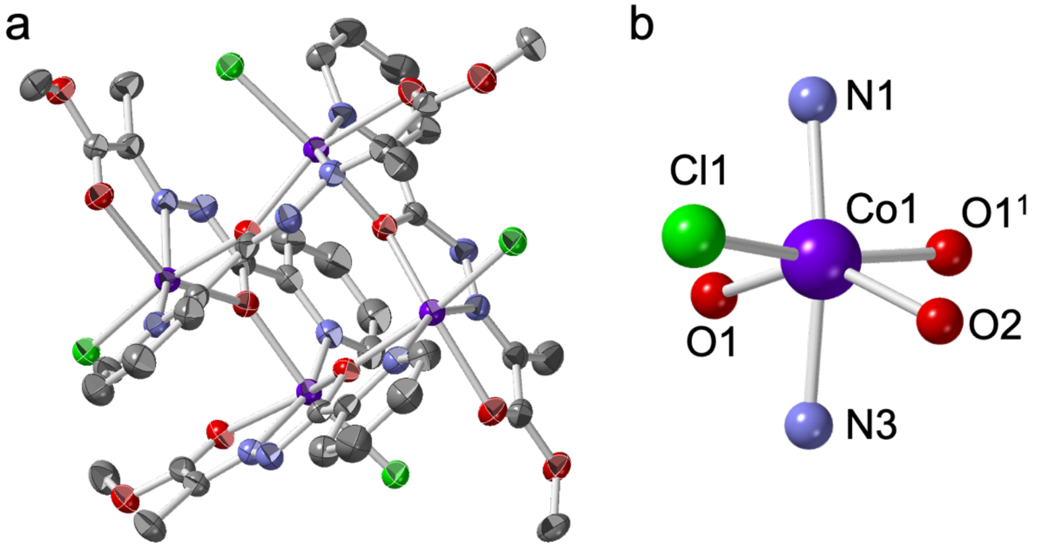

Figure S4. (**a**) ORTEP drawing with the labelling scheme of the structure of the tetrameric complex $[Co_4(L)_4Cl_4]$ (**Co4**). Ellipsoids are drawn at 30 % probability level. H atoms are omitted for clarity. (**b**) Coordination environment of the Co1 atom in compound **Co4**.

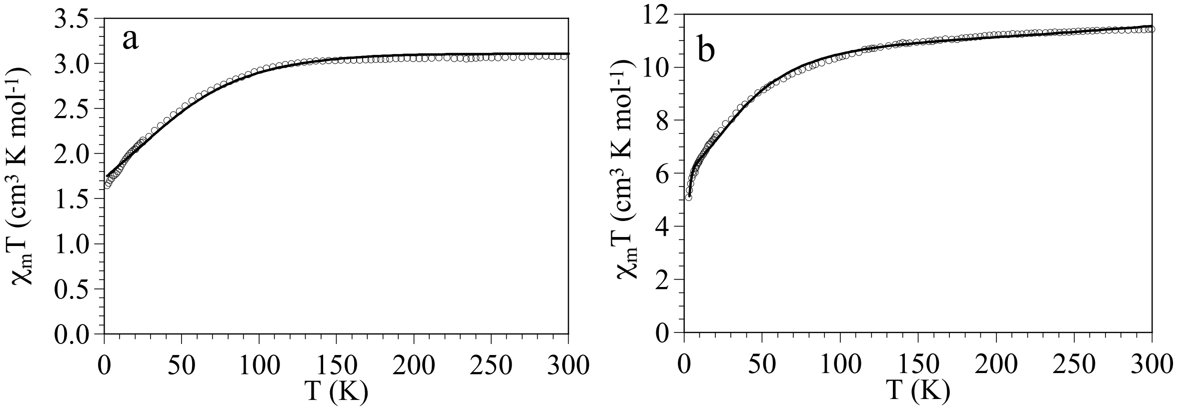

Figure S5. Magnetic properties of **Co3** and **Co4**. Thermal variation of the $\chi_m T$ product for (**a**) **Co3** and (**b**) **Co4**. Solid lines are the best fit to the model (see text).

Table S1. Crystal data for complexes **Co3** and **Co4**.

**Compound**	**Co3**	**Co4**
Empirical formula	$C_{36}H_{37}Co_3N_{12}O_{16.5}$	$C_{40}H_{40}Cl_4Co_4N_{12}O_{12}$
CCDC Number	2325183	2325184
Formula weight	1078.56	1258.36
Temperature (K)	298(2)	296(2)
Crystal system	Triclinic	Tetragonal
Space group	*P*-1 (No. 2)	$P\text{-}42_1c$ (No. 114)
a (Å)	8.0616(5)	13.0523(7)
b (Å)	10.0883(8)	13.0523(7)
c (Å)	15.0631(11)	14.8575(13)
α (º)	97.338(3)	90
β (º)	103.176(3)	90
γ (º)	109.165(3)	90
V (Å^3)	1099.03(14)	2531.2(4)
Z	1	2
ρ_{calc} (g cm^{-3})	1.603	1.651
μ (mm^{-1})	1.205	1.569
F (000)	550	1272
Crystal size (mm^3)	0.12 x 0.16 x 0.22	0.12 x 0.15 x 0.19
λ (Å) Mo Kα	0.71073	0.71073
$\theta_{min-max}$ (º)	2.3 - 25.5	2.1 - 25.1
Index ranges	$-9 \le h \le 9$<br>$-12 \le k \le 12$<br>$-18 \le l \le 18$	$-15 \le h \le 15$<br>$-15 \le k \le 15$<br>$-17 \le l \le 17$
Total data	11893	90901
Unique data	4013 (R_{int} = 0.054)	2263 (R_{int} = 0.123)
Observed Data [I > 2 σ(I)]	2977	2263
N_{ref}, N_{par}	4013, 334	2263
R	0.0485	0.0315
wR2	0.1308	0.0776
S	1.068	1.190

$w = 1/[\sigma^2(F_o^2)+(0.0675\ P)^2]$ for **Co3** and $w = 1/[\sigma^2(F_o^2)+(0.0244\ P)^2+1.7103\ P]$ for **Co4,** where $P = (F_o^2+2\ F_c^2)/3$

Table S2. Selected bond lengths (Å) for complexes **Co3** and **Co4**.

**Co3**				**Co4**	
**Atoms**	**Distance (Å)**	**Atoms**	**Distance (Å)**	**Atoms**	**Distance (Å)**
Co1-O6	1.875(3)	Co2-O4	2.047(2)	Co1-Cl1	2.3516(16)
Co1-O3	1.885(3)	Co2-O7	2.074(3)	Co1-O1	2.139(4)
Co1-N2	1.891(3)	Co2-N6	2.216(3)	Co1-O1^{2}	2.119(4)
Co1-N5	1.899(3)	Co2^{1}-O4	2.047(2)	Co1-O2	2.261(4)
Co1-N1	1.924(3)	Co2-O7^{1}	2.074(3)	Co1-N1^{2}	2.097(4)
Co1-N4	1.932(3)	Co2-N6^{1}	2.216(3)	Co1-N3	2.069(5)

Symmetry code: (1) 1-x, -y, 1-z.; (2) y, 1-x, 1-z.

Table S3. Selected Bond angles (º) for complexes **Co3** and **Co4**

**Co3**				**Co4**	
**Atoms**	**Angle (º)**	**Atoms**	**Angle (º)**	**Atoms**	**Angle (º)**
O6-Co1-O3	87.78(13)	O4-Co2-O4^{1}	180	N3-Co1-N1^{2}	156.4(2)
O6-Co1-N2	90.02(13)	O4-Co2-O7^{1}	91.90(12)	N3-Co1-O1^{2}	93.46(16)
O3-Co1-N2	93.86(13)	O4^{1}-Co2-O7^{1}	88.10(12)	N1^{i}-Co1-O1"	77.09(17)
O6-Co1-N5	92.92(12)	O4-Co2-O7	88.10(12)	N3-Co1-O1	74.07(16)
O3-Co1-N5	87.98(13)	O4-Co2-O7^{1}	91.90(12)	N1^{2}-Co1-O1	125.63(16)
N2-Co1-N5	176.60(13)	O7^{1}-Co2-O7	180	O1^{2}-Co1-O1	85.34(19)
O6-Co1-N1	88.62(13)	O4-Co2-N6	76.67(11)	N3-Co1-O2	73.74(17)
O3-Co1-N1	175.47(13)	O4^{1}-Co2-N6	103.33(11)	N1^{2}-Co1-O2	83.90(17)
N2-Co1-N1	83.39(14)	O7^{1}-Co2-N6	88.91(13)	O1^{2}-Co1-O2	85.53(15)
N5-Co1-N1	94.95(13)	O7-Co2-N6	91.09(13)	O1-Co1-O2	145.85(15)
O6-Co1-N4	175.35(13)	O4-Co2-N6^{1}	103.33(11)	N3-Co1-Cl1	96.35(13)
O3-Co1-N4	89.63(13)	O4^{1}-Co2-N6^{1}	76.67(11)	N1^{2}-Co1-Cl1	95.53(14)
N2-Co1-N4	94.01(13)	O7^{1}-Co2-N6^{1}	91.09(14)	O1^{2}-Co1-Cl1	169.02(11)
N5-Co1-N4	83.13(13)	O7-Co2-N6^{1}	88.91(13)	O1-Co1-Cl1	92.61(11)
N1-Co1-N4	94.15(13)	N6-Co2-N6^{1}	180	O2-Co1-Cl1	101.93(12)

Symmetry code: (1) 1-x, -y, 1-z.; (2) y, 1-x, 1-z.

Table S4. Selected hydrogen bonding dimensions (Å, º) for compounds **Co3** and **Co4**.

**Co3**				
**D-H···A**	**D-H**	**H···A**	**D···A**	**< D-H···A**
O7-H71-O8	0.83(3)	1.75(3)	2.576(9)	169(3)
C8-H8C-O10B	0.96	2.43	3.29(5)	149
O9-H92-O7	0.83	2.02	2.839(12)	167
O10A-H102-O2	0.83	2.51	3.34(5)	171
**Co4**				
C2-H2-Cl1	0.93	2.77	3.372(6)	123
C3-H3-Cl1	0.93	2.80	3.675(7)	156

Table S5. Continuous SHAPE measurement values of the five possible coordination geometries with coordination number six for the three cobalt centers in compounds **Co3** and **Co4**. Lowest values are indicated in bold.[S8,S9]

		**Co3**		**Co4**
**Geometry**	**Symmetry**	**Co1**	**Co2**	**Co1**
HP-6	D_{6h}	30.827	31.799	32.013
PPY-6	C_{5v}	27.797	20.909	19.322
**OC-6**	$\mathbf{O_h}$	**0.306**	**2.377**	**4.532**
TPR-6	D_{3h}	15.302	8.735	8.478
JPPY-6	C_{5v}	31.267	24.846	24.407

HP-6 = Hexagon, PPY-6 = Pentagonal pyramid, OC-6 = Octahedron, TPR-6 = Trigonal prism and JPPY-6 = Johnson pentagonal pyramid J2.

Section 3: Electrochemical Measurements

OER Measurements

Initially, the electrodes loaded with the electrocatalyst (EC) were continually scanned through cyclic voltametric (CV) measurements until a static CV plot was achieved. Then, the OER polarization curves were measured for both ECs through linear sweep voltammetry (LSV) in the potential window from 1.1 to 1.9 V (*vs*. the reversible hydrogen electrode, RHE) with a scan rate of 0.005 V s^{-1}. The current densities were normalized to the surface area of the electrode. Electrochemical impedance spectroscopy (EIS) was conducted at a constant overpotential (η) of 130 mV with the frequency ranging from 5 MHz to 100 kHz. The electrocatalytic stability of the ECs were examined by chronopotentiometric measurement as well as LSV. All polarization curves were corrected by iR compensation. The η at 10 mA cm^{-2} (η_{10}) was calculated as follows:

$$\eta_{10} = E_{10} - 1.23 \quad \text{(S1)}$$

Where E_{10} is the potential of OER at a current density of 10 mA cm^{-2} and 1.23 V is the standard redox potential of the O_2/H_2O couple.

The Tafel slope (b) was obtained using the following equation:

$$\eta = a + b \log j \quad \text{(S2)}$$

Where η is the overpotential, b is the Tafel slope and j is the current density (mA cm^{-2}).

HER Measurements

The polarization curves of HER for both ECs were measured under the potential range -0.4 to 0.0 V (*vs*. RHE) with a scan rate of 0.005 V s^{-1}. The EIS measurements were performed at constant overpotential (η) of 49 mV. Tafel analyses and stability measurements were done using similar procedure as OER. The iR-compensation was adopted to reduce the conductivity impact on HER performance.

Overall Water Splitting (OWS) Measurements

In order to investigate the real energy conversion, an OWS test was carried out in a two-electrode system where **Co4** coated on Ni foam was used as symmetric bifunctional electrocatalytic electrodes with a mass loading of 0.24 mg cm^{-2} on each electrode. Moreover, the benchmark Pt/C (cathode) ‖ RuO_2/C (anode) couple was used as reference under similar conditions. The polarization curves were measured by LSV in the potential range 1.2 to 1.7 V (*vs*. RHE) with a scan rate of 0.005 V s^{-1}. Prior to OWS test, the KOH solution was purged with N_2 for at least 30 min. The electrocatalytic OWS stability was further examined through chronoamperometric measurements for 24 h.

OER Mechanism

For the production of one O_2 molecule, 4 electrons are required to be transferred. This e-transfer may possibly happen *via* multiple steps (Equations S3-S6). The theoretical thermodynamic voltage for this multistep 4 electron oxidation processes is 1.23 V. The whole OER, consisting of 4 elementary steps can be described, in alkaline media, as follows:

Step I:	$M^* + OH^- \rightarrow M\text{-}OH^* + e^-$	(S3)
Step II:	$M\text{-}OH^* + OH^- \rightarrow M\text{-}O^* + H_2O + e^-$	(S4)
Step III:	$M\text{-}O^* + OH^- \rightarrow M\text{-}HOO^* + e^-$	(S5)
Step IV:	$M\text{-}HOO^* + OH^- \rightarrow O_2 + H_2O + e^- + M^*$	(S6)
**Overall reaction:**	$\mathbf{4\ OH^- \rightarrow O_2 + H_2O + 4\ e^-}$	

Where M* indicates the active sites of the EC. During the 4 elementary steps (S3-S6), the produced three reaction intermediates M-OH*, M-O*, and M-HOO* imply the adsorbed oxygen-containing intermediate species on the EC surface. In Step I, the oxidation of OH^- ions was initiated to form OH*, followed by deprotonation in Step II and further oxidation for HOO* generation (Step III), and finally O_2 is generated in Step IV.

HER Mechanism

The electrochemical HER is a two-electron reduction reaction of H_2O molecules occurring at the cathode surface at a theoretical thermodynamic voltage of 0 V. The mechanistic pathways for HER reaction are highly dependent on the pH of the electrolyte and follow the Volmer-Heyrovsky and Volmer-Tafel process. In general, the mechanistic pathway for HER in alkaline media can be expressed as follows:

Step I (Volmer):	$M + H_2O + e^- \rightarrow M\text{-}H_{ads} + OH^-$	(S7)
Step IIa (Heyrovsky):	$M\text{-}H_{ads} + H_2O + e^- \rightarrow M + H_2 + OH^-$	(S8)
Step IIb (Tafel):	$2\ M\text{-}H_{ads} \rightarrow 2\ M + H_2$	(S9)
**Overall reaction:**	$\mathbf{2\ H_2O + 2\ e^- \rightarrow H_2 + 2\ OH^-}$	(S10)

Here, M represents the active metal center of the EC and H_{ads} the adsorbed H atom at the active center of the EC. The Tafel slopes for the above reaction steps (Equations S7-S9) are 120, 40, and 30 mV dec^{-1}, respectively. Herein, H_{ads} is primarily generated from the cleavage of H_2O molecules in Volmer reaction (step I). In step II, H_2 molecules are produced following two different routes. One is the Heyrovsky reaction (step IIa) where combination of one proton from a H_2O molecule with the generated H_{ads} in step I generates an H_2 molecule. The other possible route is the Tafel reaction (step IIb), where an H_2 molecule is produced through direct combination of two generated H_{ads} (in step I).

Parameters Calculation

The electrochemical active surface area (ECSA) of the ECs was calculated using the following equation:

$$ECSA = C_{dl} / C_s \qquad \text{(S11)}$$

Where C_{dl} represents the double-layer capacitance and C_s indicates the ideal specific capacitance per unit area in the same electrolyte of a smooth planar surface prepared with a similar material. Herein, we have used the general specific capacitances of C_s = 0.04 mF cm^{-2} for 1.0 M KOH.

The roughness factor (R_f) was estimated by the following equation:

$$R_f = ECSA / GSA \quad (S12)$$

Where GSA is the geometric surface area of the electrode (0.126 cm^2 in our case).

The mass activity (j_m) was calculated using the following equation:

$$j_m = j / m \quad (S13)$$

Where j is the measured current density (in mA cm^{-2}) and m is the mass loading surface density of the EC (in mg cm^{-2}). In this work we have calculated the mass activity for an overpotential (η) of 250 mV for OER and 110 mV for HER.

The turnover frequency (TOF) for OER was calculated using the following equation:

$$TOF(OER) = j\ S / 4\ F\ n \quad (S14)$$

Where, j is the measured current density in A cm^{-2} at η = 240 mV, S is the electrode surface (cm^2) containing the active EC, 4 is the number of electrons transferred in OER, F is the Faraday constant (96485 C mol^{-1}) and n is the number of deposited EC moles on the working electrode.

For the HER, the TOF can be calculated using the equation:

$$TOF(HER) = I / 2\ F\ n \quad (S15)$$

Where I is the polarization curve current (in A) at η = 113 mV. Here, the factor 2 represents the required two electrons for the formation of one hydrogen molecule in HER. F and n have the same meaning as in equation S13.

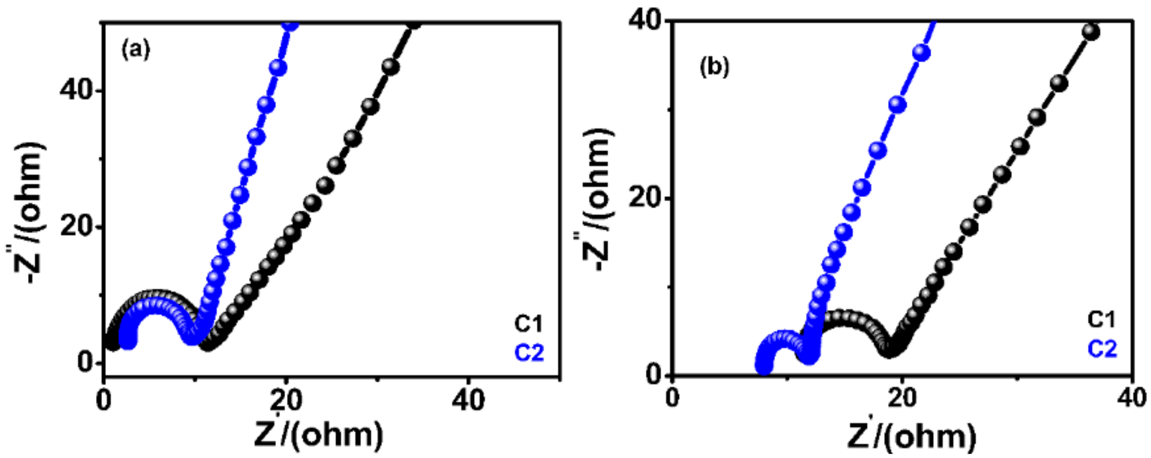

Figure S6. Electrochemical impedance spectroscopy (EIS) of **Co3** and **Co4** in 1.0 M KOH under 1600 rpm rotational speed with (**a**) η = 157 mV for OER and (**b**) η = 40 mV for HER.

Table S6. Surface parameters of the ECs during OER and HER.

**Compound**	**Reaction**	**Mass loading ($mg\ cm^{-2}$)**	**R_{ct} (Ω)**	**C_{dl} ($mF\ cm^{-2}$)**	**ECSA ($m^2\ g^{-1}$)**	**R_f**
**Co3**	OER	~ 0.20	10.4	4.5	56.2	892.8
**Co4**	OER	~ 0.20	6.9	13.9	173.7	2757.9
**Co3**	HER	~ 0.20	7.4	11.2	140.0	2222.2
**Co4**	HER	~ 0.20	3.8	19.1	238.7	3789.6

R_{ct} = Charge transfer resistance; C_{dl} = double-layer capacitance; ECSA = electrochemical active surface area; R_f = roughness factor.

Table S7. Electrocatalytic OER Performances of **Co3** and **Co4**.

ECs	E_{onset} (V)[a]	η (mV)[b]	η (mV)[c]	j (mA cm^{-2})[d]	Δη (mV)[b]	Mass activity (A g^{-1})[e]	Tafel slope (mV dec^{-1})[f]	Tafel slope (mV dec^{-1})[g]
**Co3**	1.38	220	270	228.3	+20	77.8	67.5	76.4
**Co4**	1.32	157	200	320.7	0	152.0	40.0	40.0

(a) *vs*. RHE; (b) j = 10 mA cm^{-2}; (c) j = 20 mA cm^{-2}; (d) η = 570 mV; (e) at 1.48 V; (f) before; (g) after.

Table S8. Electrocatalytic HER Performances of the **Co3** and **Co4**.

ECs	E_{onset} (V)[a]	η (mV)[b]	η (mV)[c]	j (mA cm^{-2})[d]	Δη (mV)[b]	Mass activity (A g^{-1})[e]	Tafel slope (mV dec^{-1})[f]	Tafel slope (mV dec^{-1})[g]
**Co3**	53.3	70.2	105.3	21.0	+5	221.2	59.2	62.8
**Co4**	34.1	39.8	64.5	50.0	0	500.0	40.4	37.3

(a) *vs*. RHE; (b) j = 10 mA cm^{-2}; (c) j = 20 mA cm^{-2}; (d) η = 107 mV; (e) at 150 mV; (f) before; (g) after.

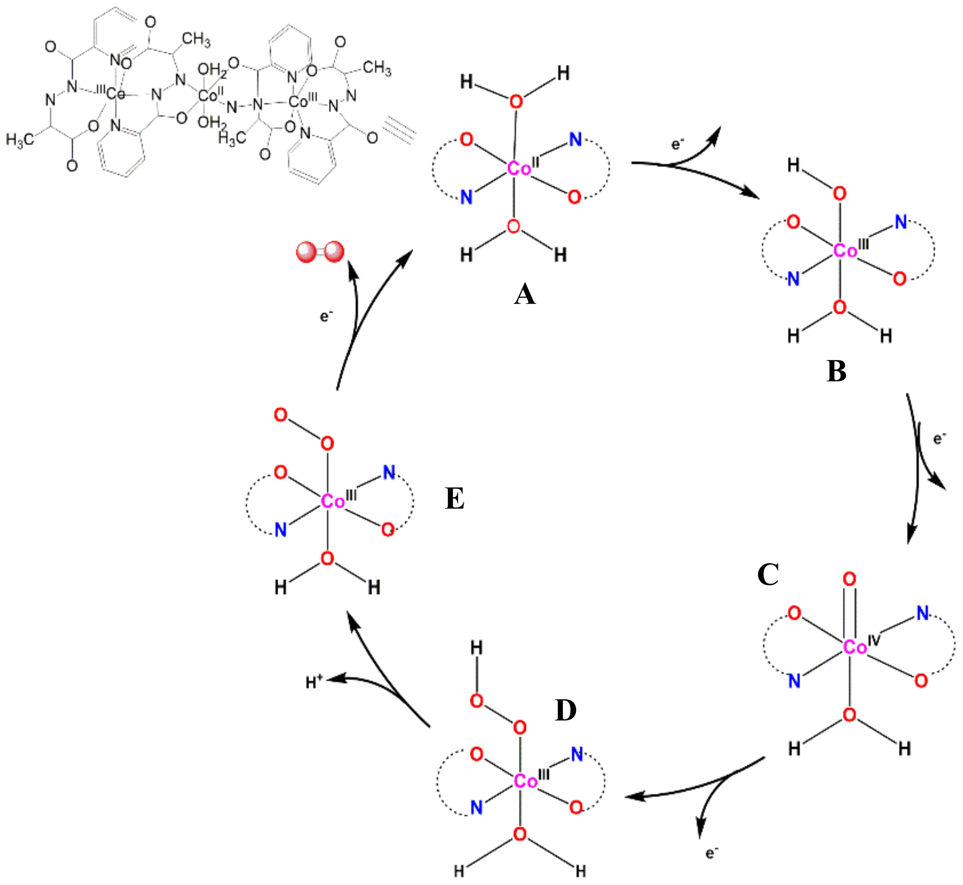

Scheme S1. Probable catalytic cycle for the production of oxygen mediated by **Co3**.

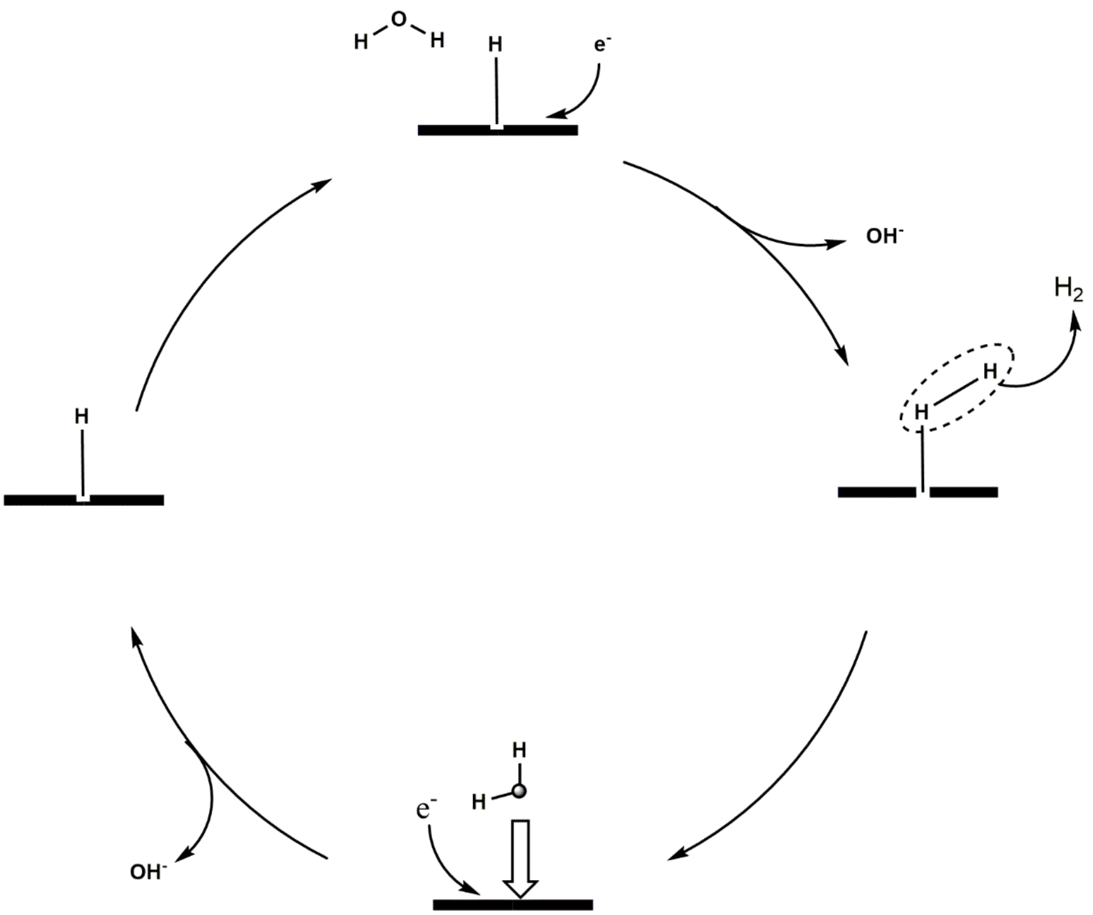

Scheme S2. Proposed catalytic cycle for the hydrogen evolution reaction mediated by the catalysts.

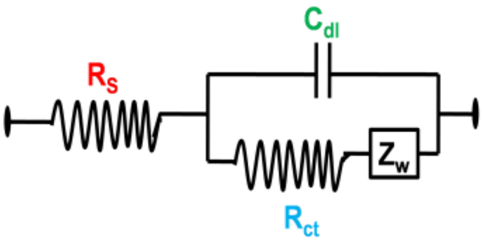

Scheme S3. Randles circuit of the electrochemical cell used for the electrocatalysis measurements (R_s, R_{ct} and C_{dl} are the solution resistance, charge transfer resistance and double layer capacitance, respectively).

Section 4: Spectroscopic Characterization

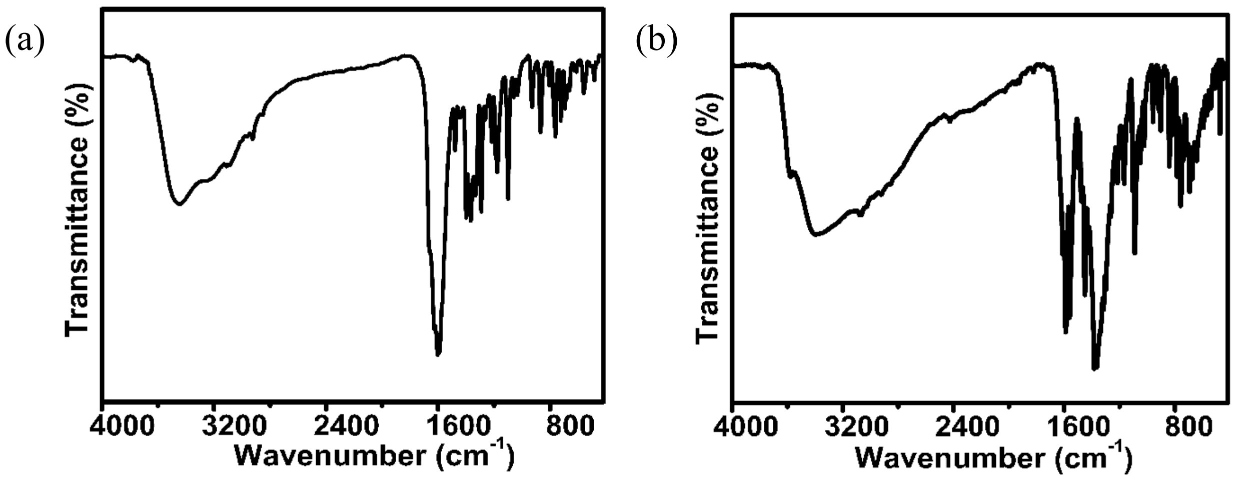

Figure S7. IR spectra of (**a**) **Co3** and (**b**) **Co4**.

Evidence for stability of the electrocatalysts

The stability of both ECs during the catalytic cycles was confirmed by different techniques as follow:

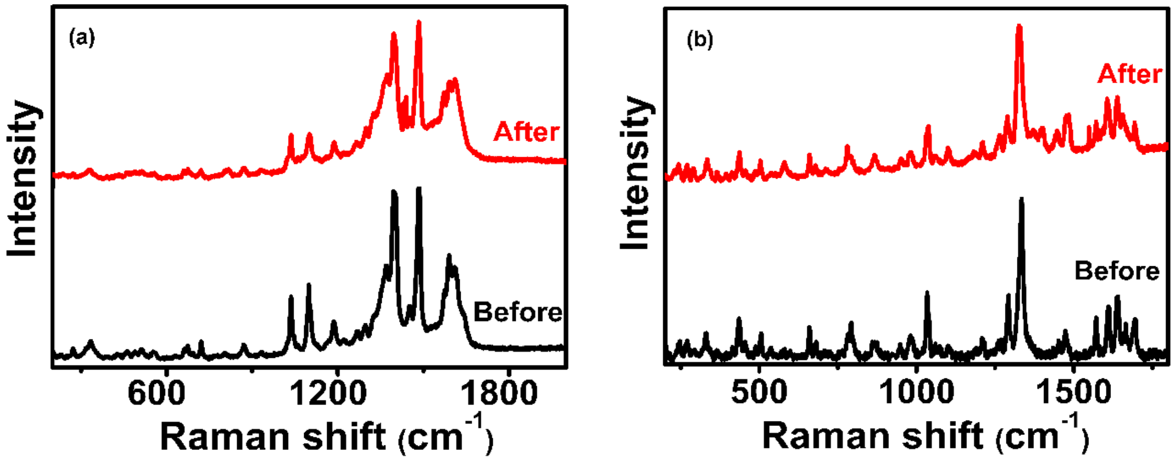

Figure S8. Raman spectra of (**a**) **Co3** (left) and (**b**) **Co4** (before and after OER).

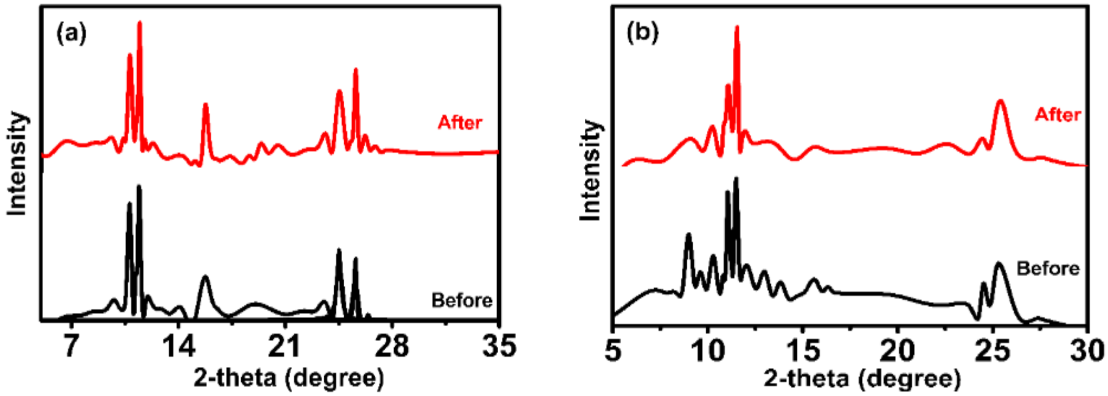

Figure S9. X-ray powder diffractograms of (**a**) **Co3** and (**b**) **Co4** (before and after OER).

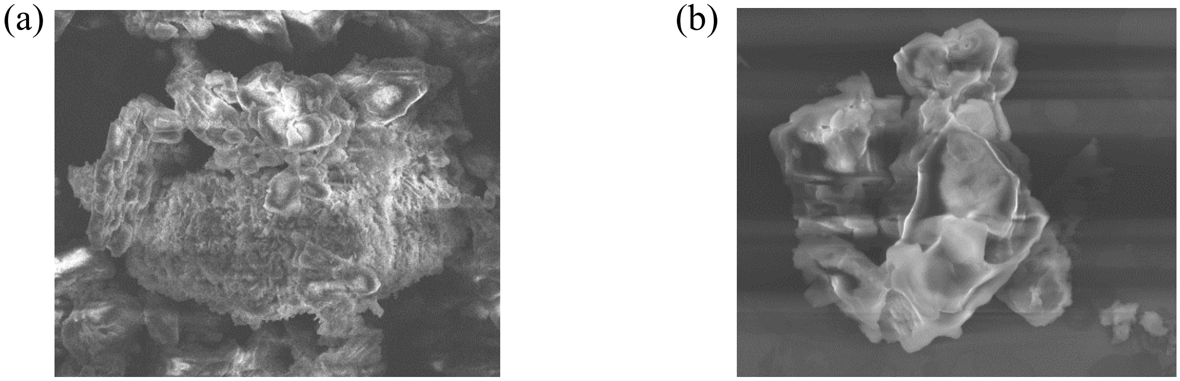

Figure S10. SEM images of **Co3** (**a**) before and (**b**) after OER.

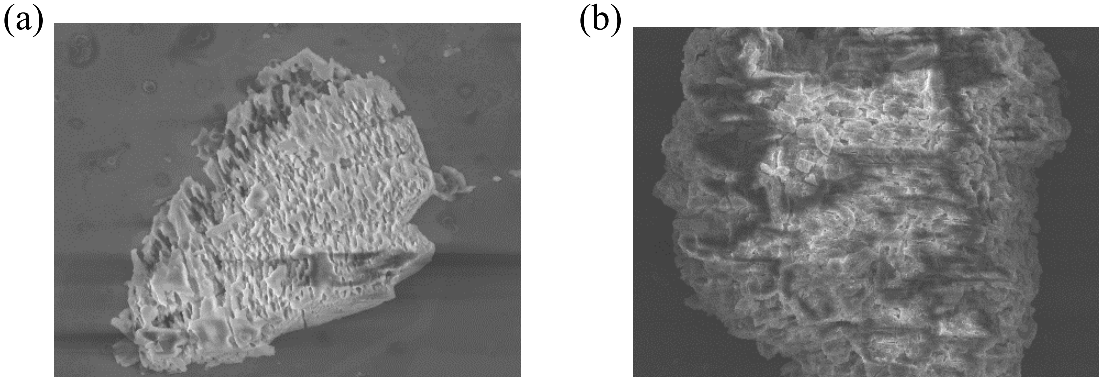

Figure S11. SEM images of **Co4** (**a**) before and (**b**) after OER.

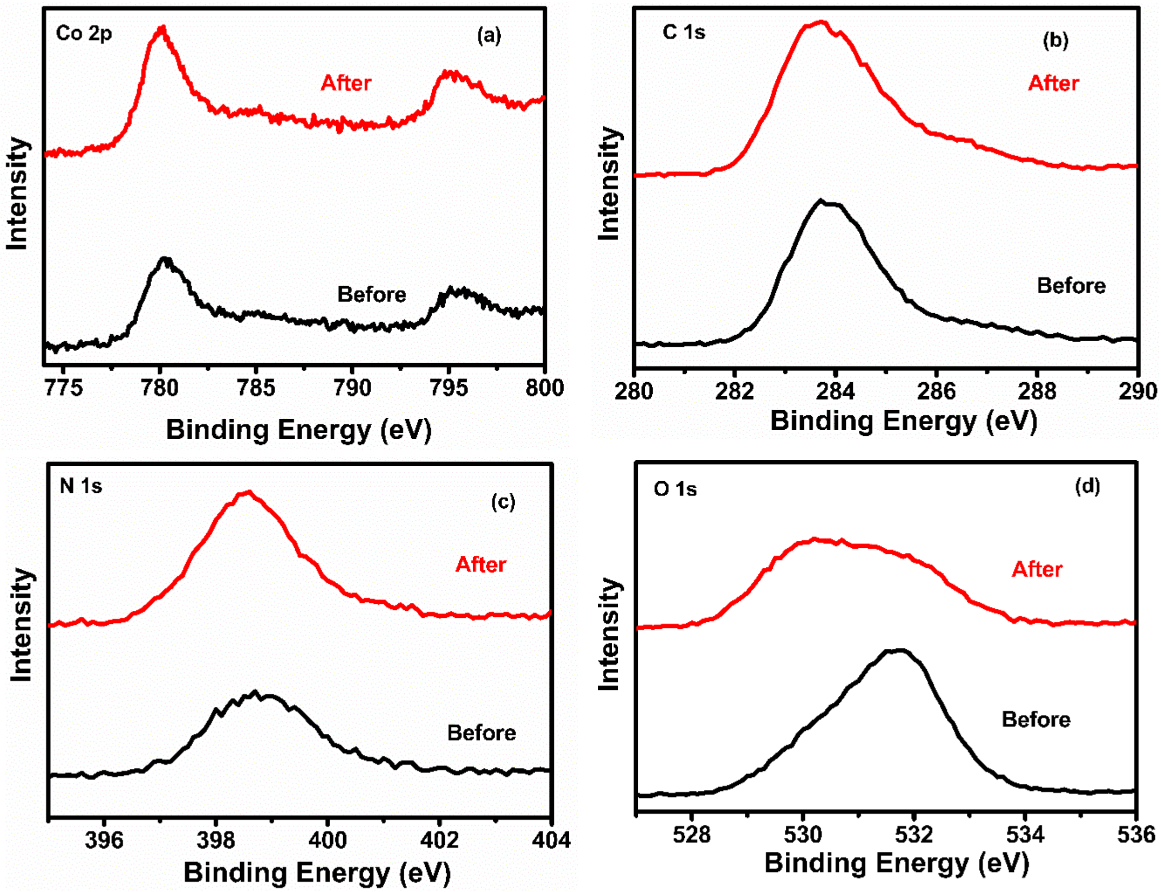

Figure S12. XPS spectra of Co3 before and after OER; (a) the Co 2p, (b) C 1s, (c) N 1s and (d) O 1s XPS core level spectrum.

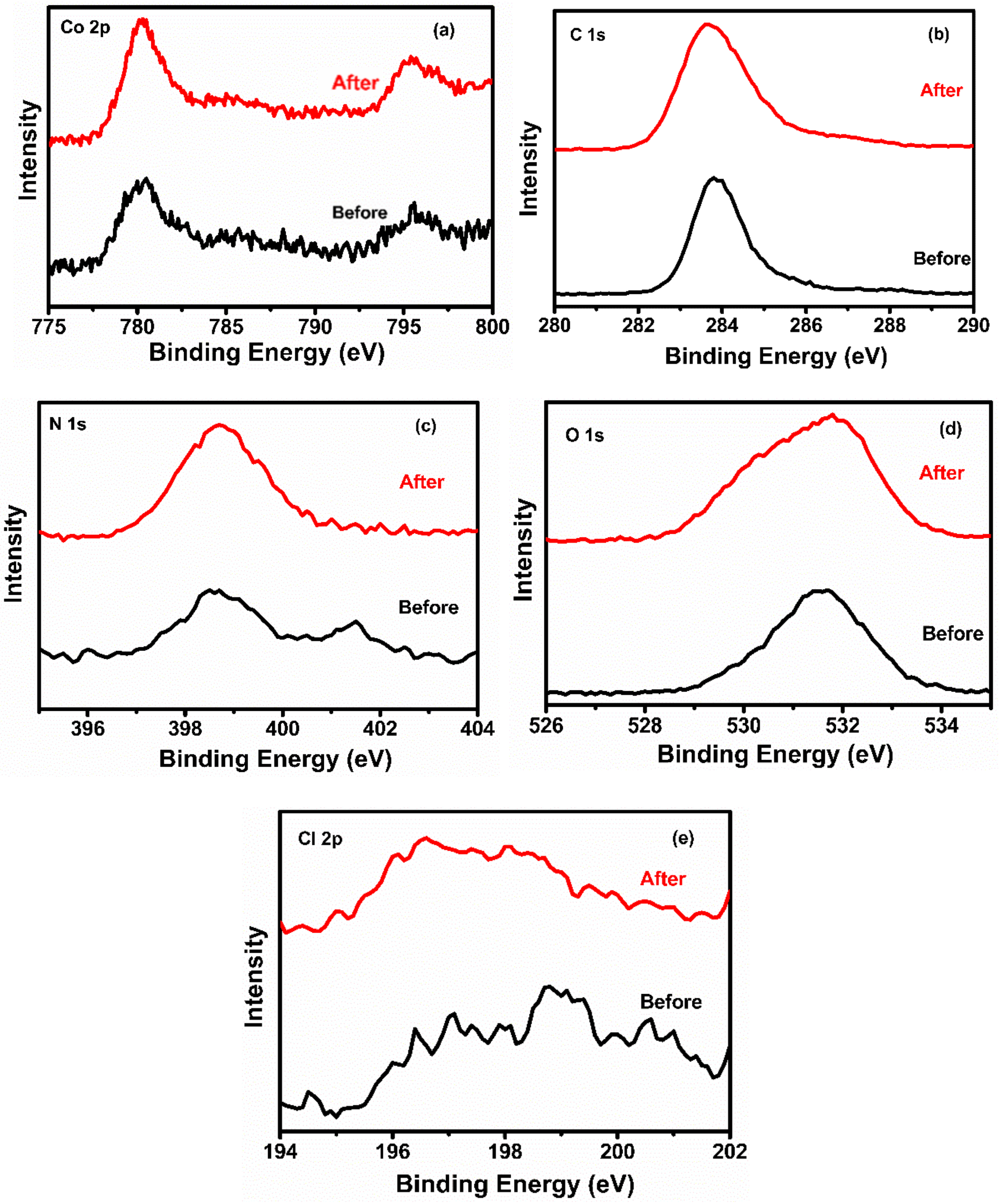

Figure S13. XPS spectra of **Co4** before and after OER; (a) the Co 2p, (b) C 1s, (c) N 1s, (d) O 1s and (e) Cl 2p XPS core level spectrum.

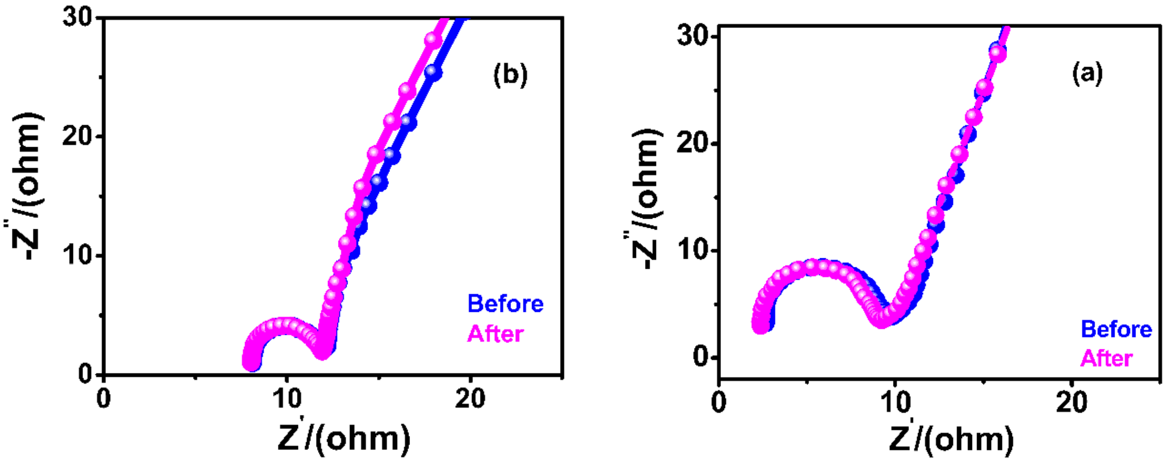

Figure S14. EIS measurements of Co4 (a) OER and (b) HER stability test.

References

(S1) APEX4, SAINT and SADABS; Bruker AXS Inc., Madison, WI 2019-2022.

(S2) Sheldrick G. M., *Acta Cryst.* **2015**, *A71*, 3.

(S3) Sheldrick G. M., *Acta Cryst.* **2015**, *C71*, 3.

(S4) Farrugia L. J., *J. Appl. Cryst.* **2012**, *45*, 849.

(S5) Keller E., SCHAKAL, University of Freiburg, Germany 1999.

(S6) Crystalmkaker, version 10.8.3, crystalmaker software Ltd.

(S7) Bain, G. A.; Berry, J. F. Diamagnetic Corrections and Pascal's Constants. *J. Chem. Educ.* **2008**, *85* (4), 532.

(S8) Alvarez, S. Distortion Pathways of Transition Metal Coordination Polyhedra Induced by Chelating Topology. *Chem. Rev.* **2015**, *115* (24), 13447–13483.

(S9) Alvarez, S.; Avnir, D.; Llunell, M.; Pinsky, M. Continuous Symmetry Maps and Shape Classification. The Case of Six-Coordinated Metal *New J. Chem.* **2002**, *26* (8), 996–1009.
